# A novel protein encoded by circMAPK1 inhibits progression of gastric cancer by suppressing activation of MAPK signaling

**DOI:** 10.1186/s12943-021-01358-y

**Published:** 2021-04-09

**Authors:** Tianlu Jiang, Yiwen Xia, Jialun Lv, Bowen Li, Ying Li, Sen Wang, Zhe Xuan, Li Xie, Shengkui Qiu, Zhongyuan He, Linjun Wang, Zekuan Xu

**Affiliations:** 1 Division of Gastric Surgery, Department of General Surgery, The First Affiliated Hospital of Nanjing Medical University, 300 Guangzhou Road, Nanjing, 210029 Jiangsu Province People’s Republic of China; 2 Department of General Surgery, The Second Affiliated Hospital of Nantong university, Nantong, 226001 Jiangsu Province People’s Republic of China; 3 Jiangsu Key Lab of Cancer Biomarkers, Prevention and Treatment, Collaborative Innovation Center for Cancer Personalized Medicine, Nanjing Medical University, Nanjing, 210029 Jiangsu Province People’s Republic of China

**Keywords:** Gastric cancer, Circular RNA, Coding capacity, MAPK pathway

## Abstract

**Background:**

A novel type of noncoding RNA, circRNA has been reported to participate in the occurrence and development of diseases through many mechanisms. The MAPK pathway is a common signal transduction pathway involved in cell proliferation, inflammation and apoptosis and plays a particularly important role in cancers. However, the role of circRNAs related to the MAPK pathway in gastric cancer has not been explored.

**Methods:**

A bioinformatics analysis was performed to profile and identify the circRNAs involved in the MAPK pathway in gastric cancer. The tumor-suppressive role of circMAPK1 was confirmed both in vitro and in vivo. Mass spectrometry, Western blot and immunofluorescence staining assays were used to validate the existence and expression of MAPK1–109aa. The molecular mechanism of circMAPK1 was investigated by mass spectrometry and immunoprecipitation analyses.

**Results:**

In this study, we identified that circMAPK1 (hsa_circ_0004872) was downregulated in gastric cancer tissues compared with adjacent normal tissues. Importantly, lower circMAPK1 expression predicted poor survival in GC patients. CircMAPK1 inhibited the proliferation and invasion of gastric cancer cells in vitro and in vivo. Next, we found that circMAPK1 encoded a novel protein with 109 amino acids in length. Through a series of functional experiments, we confirmed that circMAPK1 exerted a tumor-suppressing effect via the encoded protein MAPK1–109aa. Mechanistically, the tumor suppressor MAPK1–109aa inhibited the phosphorylation of MAPK1 by competitively binding to MEK1, thereby suppressing the activation of MAPK1 and its downstream factors in MAPK pathway.

**Conclusions:**

Our study revealed that circMAPK1 inhibits the malignant biological behavior of gastric cancer cells through its encoded protein MAPK1–109aa. More importantly, circMAPK1 is a favorable predictor for gastric cancer patients and may provide a new therapeutic target in the treatment of gastric cancer.

**Supplementary Information:**

The online version contains supplementary material available at 10.1186/s12943-021-01358-y.

## Background

Gastric cancer (GC) is one of the most common malignancies with high morbidity and mortality worldwide, especially in East Asian countries such as China, Japan and Korea [1]. Gastric tumorigenesis is a multistage, slowly progressing and multifactorial pathological process [2]. The proven high-risk factors for GC include having *Helicobacter pylori* infection, excessive intake of salt and nitrates, obesity and blood group A [3]. In addition, genetic mutations, epigenetic changes, and aberrant molecular signaling pathways are also involved in the development and metastasis of GC [4]. Because early GC lacks specific symptoms, most GC patients are diagnosed at the intermediate or terminal stage and have poor prognosis [5]. It is urgent to identify the gene expression patterns and biomarkers in GC to advance GC research and improve patient survival.

Circular RNA (circRNA) is a special kind of noncoding RNA molecule that has recently become a hotspot in the field of noncoding RNA [6]. In contrast to traditional linear RNAs, circRNAs have a closed loop structure without a 5′ cap or a 3′ poly A tail [7]. The expression of circRNAs is more stable than linear RNAs, and the molecules are not as easily degradable. With the development of high-throughput sequencing technology and bioinformatic analysis, an increasing number of circRNAs have been found to regulate cellular proliferation, migration, invasion, apoptosis and differentiation [8, 9]. The great majority of circRNAs have been found to perform their biological functions by acting as microRNA (miRNA) sponges or binding to proteins [10]. A few studies have also suggested that circRNAs containing internal ribosome entry sites (IRESs) or extensive methyl modification sites have the potential to affect physiological behaviors by encoding proteins [11, 12]. For instance, circβ-catenin promotes liver cancer cell growth through activation of the Wnt pathway by encoding β-catenin-370aa [13]. SHPRH-146aa is encoded by circ-SHPRH, which can protect full-length SHPRH from degradation by the ubiquitin proteasome and inhibit glioma tumorigenesis [14]. Recently, the role of circRNAs as the sponge of miRNA and protein in GC has been well established [15–17]. However, whether circRNAs could regulate the tumorigenesis and development of GC by encoding proteins remains unknown.

The mitogen-activated protein (MAP) kinase signaling cascade Ras-Raf-MEK-MAPK signal transduction pathway is an important evolutionarily conserved signaling pathway [18]. As the classic cellular phosphorylation cascade, it has been reported to be involved in a large variety of cellular and physiological processes essential to life [19, 20]. When extracellular stimulating factors such as cytokines, neurotransmitters, and hormones bind to transmembrane receptors, the inactive Ras-GDP in the plasma membrane is converted into active Ras-GTP. To activate downstream members of the MAPK pathway, Ras-GTP stimulates the formation of an active homodimer or heterodimer consisting of A-Raf, B-Raf, and C-Raf through a complex process [21]. The Raf enzymes are serine/threonine protein kinases that catalyze the phosphorylation and activation of dual specificity mitogen-activated protein kinase kinase 1 (MEK1) and MEK2, where MEK activates MAPK [22]. MEK1/2 sequentially phosphorylates two sites on MAPK1/2: Y204/187 and T202/185 [23]. Once the two residues are phosphorylated, MAPK1/2 is activated to catalyze many cytoplasmic and nuclear substrates, including transcription factors and regulatory molecules [18, 24]. Overall, this signaling pathway can control cell growth, cell proliferation, cell survival, differentiation, immune response, metabolism, nervous system function and transcription through a series of phosphorylation reactions [25]. However, circRNAs related to the MAPK pathway are under-researched.

In this study, we identified a circRNA, named circMAPK1, derived from the MAPK1 gene by analyzing RNA sequencing data from various databases. The expression of circMAPK1 decreased in GC tissues and cells, which was related to a good prognosis in GC patients. Functional investigations showed that circMAPK1 suppressed GC cell growth in vitro and in vivo. Subsequent studies demonstrated that circMAPK1 encoded a novel 109-amino acid (aa) MAPK1 isoform. Functionally the opposite to MAPK1, MAPK1–109aa decreased MAPK1 phosphorylation by competing with MEK1, suppressed the activation of MAPK1 and its downstream factors, thereby inhibited the proliferation and invasion of GC cells. Our results indicate MAPK1–109aa, an inhibitor of the MAPK1 pathway, as an effective diagnostic tool and treatment target for GC.

## Methods

### Patients and tissues samples

A total of 80 paired human GC and adjacent non-tumor tissues were collected from 2014 to 2015 at the Gastrointestinal center, the First Affiliated Hospital of Nanjing Medical University. This study was approved by the Medical Ethics Committee of the First Affiliated Hospital of Nanjing Medical University, and the written informed consents were provided by all participant priors. Clinicopathological features including age, gender, tumor size, tumor site, lymph node metastasis, TNM stage (according to American Joint Committee on Cancer classification, AJCC) and blood vessel invasion are shown in Table 1.

**Table 1:** Correlation between circMAPK1 expression and the clinicopathologic parameter of 80 GC patients

Clinicopathologic parameter	Number	Number of patients		***p*** value
		circMAPK1^**low**^	circMAPK1^**high**^	
**Age**				
< 60 years	30	18	12	0.248
≥ 60 years	50	22	28	
**Gender**				
Male	61	33	28	0.293
Female	19	7	12	
**Tumor size**				
< 3 cm	20	15	15	0.019*
≥ 3 cm	60	25	35	
**Tumor site**				
Proximal	36	17	19	0.822
Non-proximal	44	23	21	
**Lymph node metastasis**				
N0	28	19	9	0.034*
N1-N3	52	21	31	
**TNM stage**				
I-II	33	22	11	0.022*
III	47	18	29	
**Blood vessel invasion**				
Negative	61	32	29	0.600
Positive	19	8	11	

* *p* < 0.05, ** *p* < 0.01

### Cell culture and treatment

The human GC cell lines BGC-823, SGC-7901, MGC-803, MKN-45, HGC-27, and AGS were used. Normal stomach mucosa epithelium cell line GES-1 was purchased from the Cell Center of Shanghai Institutes for Biological Sciences. The human gastric cell line AGS was cultured in F12K medium, while the rest of the cells were cultured in RPMI 1640 medium. The HEK-293 T cell line was purchased from Type Culture Collection of the Chinese Academy of Sciences (Shanghai, China) and was cultured in DMEM medium. Both media were supplemented with 10% foetal bovine serum (Invitrogen) and 1% penicillin/streptomycin (Gibco, USA). Cells were incubated at 37 °C in a humidified atmosphere of 5% CO2.

### Plasmid, siRNA, lentiviral construction and stable transfection

Human circMAPK1 over-expressing vector and the control plasmid were purchased from Genelily Biotechnology Company (Shanghai, China). The expression of circMAPK1 was transiently silenced by small interference RNAs (siRNAs) specific to human circMAPK1, which were generated by GenePharma Corporation (Shanghai, China). Transfection was carried out using Lipofectamine 3000 (Invitrogen) according to the manufacturer’s instructions. After transfection for 48 h, the transfection efficiency of cells in each group was assessed by real-time PCR analysis. The lentivirus vector containing shRNAs targeting circMAPK1 were generated by GenePharma (Shanghai, China). Stable cell lines were obtained by selection with puromycin.

**Table:** 

RT-PCR primers	Forward primer (5′ to 3′)	Reverse primer (5′ to 3′)	Amplified products (bps)
circMAPK1 (divergent primers)	TCAAGATCTGTGACTTTGGCCT	GGTGCTCAAAGGGGCTGATT	187
circMAPK1 (convergent primers)	ACTGCGCTTCAGACATGAGA	TGCTGAGGTGTTGTGTCTTCA	143
circ0006203	TCCCCATCACAAGAAGACCTGA	TGGTGTAGCCCTTTGGAGTCA	131
circ0008870	CAGCTAACGTTCTGCACCGT	GGTGCTCAAAGGGGCTGATT	133
MAPK1	GCACCAACCATCGAGCAAAT	CTTGAGGTCACGGTGCAGAA	179
GAPDH	GTCAAGGCTGAGAACGGGAA	AAATGAGCCCCAGCCTTCTC	158

Full material and methods were described in Additional file 4.

## Results

### Bioinformatics analysis of circRNAs related to the MAPK pathway in human cell lines

**Fig. 1:**
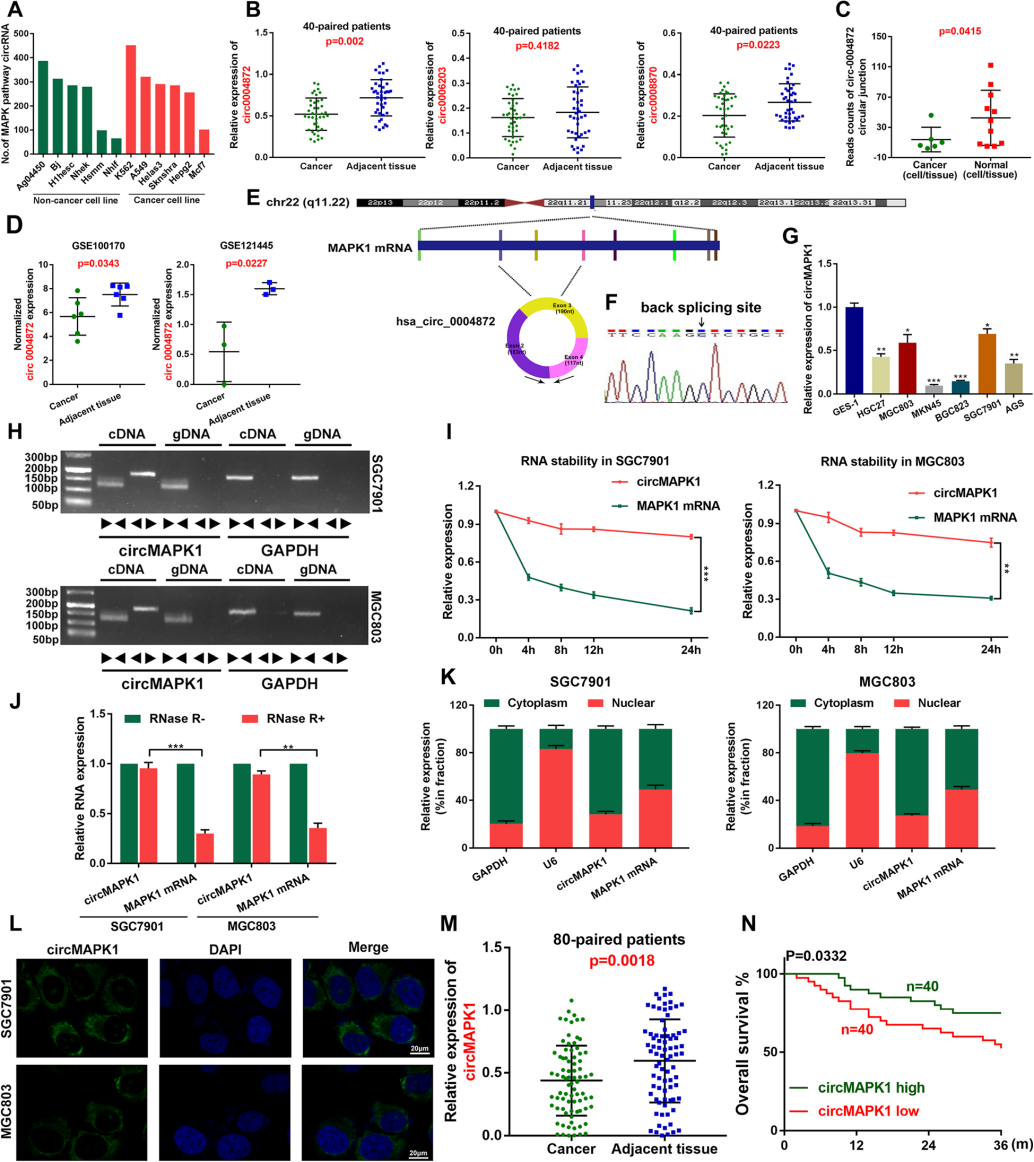
Identification and characteristics of circMAPK1 in GC. **a** The number of MAPK pathway-related circRNAs in human cell lines. **b** The expression of hsa_circ_0004872, hsa_circ_0006203 and hsa_circ_0008870 in the 40 paired GC and adjacent tissues by qRT-PCR. **c** Circular junction reads of hsa_circ_0004872 in normal and cancerous cells/tissues from Jeck2013, Salzman2013 and Rybak2015 studies in circBase. Normal cells/tissues include parietal lobe, temporal lobe, diencephalon, occipital lobe, frontal cortex, cerebellum, Hs68 RNase, A549, Ag04450, Bj, Gm12878. Cancerous cells/tissues include Helas3, Hepg2, Huvec, K562, Mcf7, Nhek, Sknshra. **d** The normalized expression of hsa_circ_0004872 in GSE100170 database and GSE121445 database. **e** The schematic diagram of circMAPK1 (hsa_circ_0004872) arose from exon 2,3,4 of the MAPK1 gene. **f** Sanger sequencing was conducted to confirm head-to-tail splicing. **g** The expression level of circMAPK1 in a series of cultured GC cell lines (GES-1, HGC27, MGC803, MKN45, BGC823, SGC7901, AGS) was analyzed by real-time PCR. **h** The divergent primers detected circMAPK1 in cDNA but not in gDNA, GAPDH was used as a negative control. **i** In SGC7901 and MGC803 cells treated with Actinomycin D, relative RNA levels of circMAPK1 and MAPK1 mRNA were measured in different time points. **j** Real-time PCR analysis of circMAPK1 and linear MAPK1 mRNA after treatment with RNase R in SGC7901 and MGC803 cells showed that circMAPK1 was resistant to RNase R treatment. **k** The nuclear mass separation assay and **l** FISH showed that the sub-cellular distribution of circMAPK1 was mostly present in the cytoplasm. **m** Relative circMAPK1 expression in additional 80 paired GC and adjacent tissues. **N** Overall survival analysis based on circMAPK1 expression in 80 GC patients. Graph represents mean ± SD; *p < 0.05, **p < 0.01, and ***p < 0.001

Since the MAPK signaling pathway plays an important pathological role in tumorigenesis and progression [26], we first analyzed circRNAs derived from MAPK pathway-related genes according to the circRNA sequencing data from the online database circBase (Additional file 1: Table S1). We then counted the number of MAPK pathway-related circRNAs in six noncancer cell lines and six cancer cell lines and found that most of these cell lines can express hundreds of MAPK pathway-related circRNAs (Fig. 1a), which implies the importance of MAPK pathway-derived circRNAs in human diseases.

We then focused on the fifteen circRNAs arising from MAPK1 due to the key role of MAPK1 in the MAPK pathway (Additional file 2: Table S2). Among the circRNAs originating from MAPK1, circ-0006203, circ-0004872 and circ-0008870 were widely detected in more than three independent sequencing datasets. Using quantitative real-time PCR (qRT-PCR), we tested the expression levels of these three circRNAs in 40 GC and paired adjacent tissues from GC patients (Fig. 1b). The results showed that the expression of circ-0004872 was the most significantly changed. Besides, we analyzed the reads of circ-0004872 junction in Jeck2013, Salzman2013 and Rybak2015. The results showed that the reads of circ-0004872 in cancerous tissues / cells were significantly lower than those in normal tissues / cells (Fig. 1c). Furthermore, the down-regulation of circMAPK1 in GC was also confirmed in GSE121445 and GSE100170 database (Fig. 1d). These data suggested the potential suppressive effects of circ-0004872, thus prompting us to further investigate its role in GC malignancy.

### Identification of circMAPK1 and the clinical features of circMAPK1

Circ-0004872 (termed “circMAPK1” in this study) was derived from exons 2–4 of the MAPK1 gene and formed a sense-overlapping circular transcript of 490 nt (Fig. 1e). Furthermore, head-to-tail splicing of the amplified circMAPK1 was confirmed by Sanger sequencing (Fig. 1f).

Next, we investigated the expression level of circMAPK1 in GC cell lines. As shown in Fig. 1g, circMAPK1 was significantly downregulated in cultured GC cell lines (MKN45, BGC823, SGC7901, MGC803, HGC27) compared with a normal gastric mucosal epithelial cell line (GES-1).

To characterize circMAPK1, we designed divergent and convergent primers. First, complementary DNA (cDNA) and genomic DNA (gDNA) extracted from SGC7901 and MGC803 cells were used as templates, and the gel electrophoresis results showed that circMAPK1 was amplified only from cDNA but not gDNA (Fig. 1h), indicating that the loop structure of circMAPK1 is generated from back-splicing. We then further evaluated the stability and localization of circMAPK1. Following actinomycin D (an inhibitor of transcription) treatment, the half-life of circMAPK1 was obviously longer than that of linear MAPK1 (Fig. 1i). Compared with the linear form of MAPK1 mRNA, circMAPK1 was resistant to RNase R degradation, which also suggested that circMAPK1 is highly stable (Fig. 1j). Subsequent qRT-PCR analysis of cell fractions (Fig. 1k) and fluorescence in situ hybridization (FISH) (Fig. 1l) assays showed that circMAPK1 was predominantly localized in the cytoplasm rather than the nucleus.

Since circMAPK1 is stably expressed in GC cell lines, we speculated that circMAPK1 may be a suitable marker for the diagnosis or prognosis of GC. We re-examined the expression of circMAPK1 in GC tissues and paired adjacent nontumor tissue samples from 80 patients by qRT-PCR (Fig. 1m). Among them, 70% of samples (*n* = 56) exhibited lower expression in cancer tissues than in matched noncancerous tissues (Additional file 3: Fig. S1A). Furthermore, we collected clinical data on the aforementioned patients and found that the expression level of circMAPK1 was significantly correlated with GC tumor size, lymphatic invasion and TNM stage (Table 1). Additionally, we generated an overall survival (OS) curve using Kaplan-Meier survival analysis and obtained survival information for the patients we followed up previously. Patients who had higher levels of circMAPK1 in their GC tissues had significantly longer overall survival (Fig. 1n).

Overall, circMAPK1 was demonstrated to be a stably expressed circRNA that could be used as an effective diagnostic and prognostic marker, which is worthy of further study.

### CircMAPK1 inhibits the proliferation and migration of GC cells in vitro

**Fig. 2:**
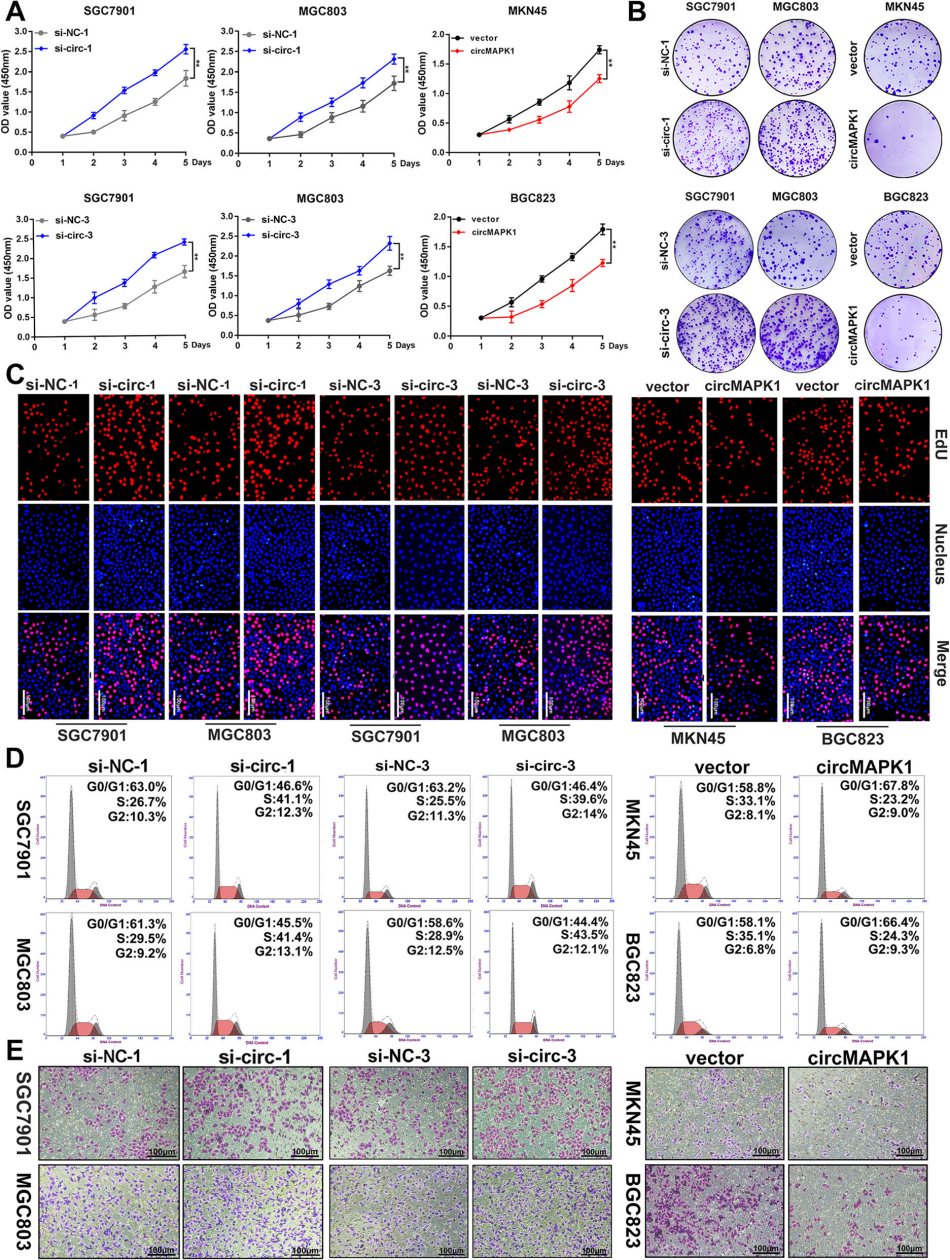
The suppressive function of circMAPK1 in vitro. **a** CCK-8 assay was performed to evaluate proliferation ability of cells after upregulating or downregulating circMAPK1 in GC cells. **b** Colony formation assay was performed to evaluate proliferation ability of cells after upregulating or downregulating circMAPK1 in GC cells. **c** EdU assay was performed to evaluate proliferation ability of cells after upregulating or downregulating circMAPK1 in GC cells. Scale bar: 100 μm. **d** The effect of circMAPK1 on modulating cell cycle progression was evaluated by flow cytometry assay. **e** The effect of circMAPK1 on cell migration was examined by Transwell assay. Scale bar: 100 μm. Graph represents mean ± SD; *p < 0.05, **p < 0.01, and ***p < 0.001

To investigate the biological function of circMAPK1 in GC, small interfering RNAs (siRNAs) specifically targeting the back-splice region of circMAPK1 was designed (Additional file 3: Fig. S1B and Fig. S1C). We transferred the siRNAs into SGC7901 and MGC803 cells because the expression of circMAPK1 is relatively high in these cell lines. qRT-PCR showed that among the three small interfering RNAs we designed, siRNA-1 and siRNA-3 effectively decreased the level of circMAPK1 in SGC7901 and MGC803 cells, while they did not affect the mRNA level of MAPK1 (Additional file 3: Fig. S1D and S1E). Subsequent CCK8 assay (Fig. 2a), colony formation assay (Fig. 2b and Additional file 3: Fig. S2A) and EdU assay (Fig. 2c and Additional file 3: Fig. S2B) showed that the proliferation ability of GC cells was significantly enhanced by silencing circMAPK1. Flow cytometry was conducted to monitor cell cycle progression. We found that the decrease in circMAPK1 increased the number of GC cells in S phase (Fig. 2d and Additional file 3: Fig. S2C), indicating that circMAPK1 inhibited the cell cycle progression of tumor cells. Further Transwell (Fig. 2e and Additional file 3: Fig. S2D) and wound healing assays (Additional file 3: Fig. S1G and S1H) showed that sicircMAPK1 promoted the migration of GC cells.

Furthermore, MKN45 and BGC823 cells were transfected with the circMAPK1 plasmid as the expression of circMAPK1 is relatively low in these cell lines. The overexpression efficiency was confirmed by qRT-PCR (Additional file 3: Fig. S1F). We found that overexpression of circMAPK1 inhibited the proliferation and migration of GC cells. CCK8 (Fig. 2a), colony formation (Fig. 2b and Additional file 3: Fig. S2A) and EdU (Fig. 2c and Additional file 3: Fig. S2B) assays revealed that overexpression of circMAPK1 markedly impaired the proliferation abilities of GC cells. Similarly, the number of GC cells in S phase was significantly reduced when circMAPK1 was overexpressed (Fig. 2D and Additional file 3: Fig. S2C). The suppression of cell migration was also verified by Transwell (Fig. 2e and Additional file 3: Fig. S2D) and wound healing assays (Additional file 3: Fig. S1I and S1J).

Collectively, these results suggest that circMAPK1 plays a critical role in the proliferation and migration of GC cells.

### CircMAPK1 suppresses tumorigenesis and metastasis of GC in vivo

**Fig. 3:**
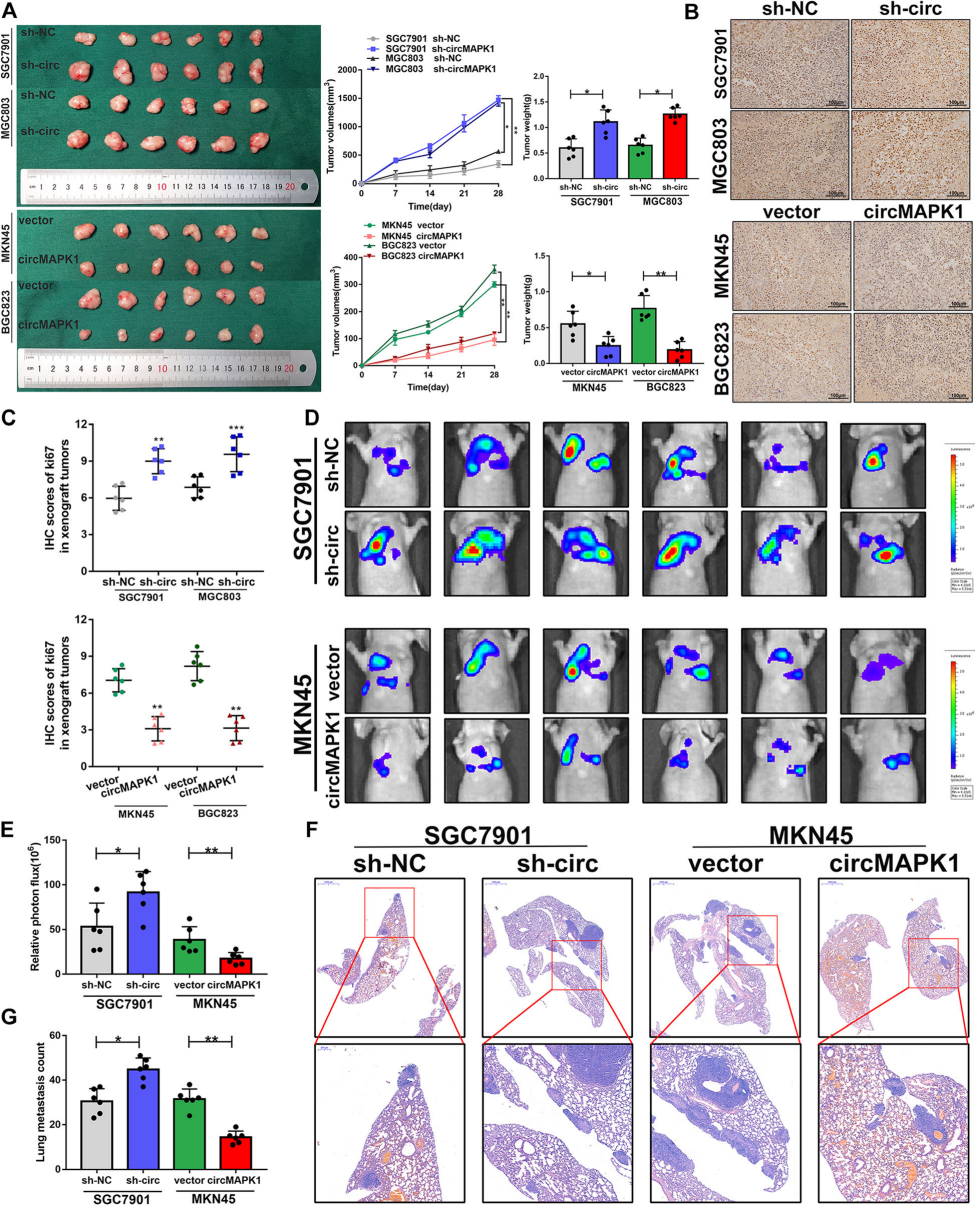
The suppressive function of circMAPK1 in vivo. **a** Left panel: The designated tumor cells were injected subcutaneously into nude mice, and xenograft tumors were collected 28 days later. Middle panel: Tumor volume was measured and calculated every week. Right panel: Tumor weight was measured after 28 days. **b** The tumor tissues were stained with Ki67 antibody. Scale bar: 100 μm. **c** IHC scores of Ki67 in respective xenograft tumor tissues. **d** Bioluminescence imaging and **e** Quantification of lung metastasis after injecting tumor cells into the tail vein of nude mice. **f** Representative images and **G** Quantification of H&E staining of the mouse lung metastasis. Scale bar: 1000 μm. Graph represents mean ± SD; *p < 0.05, **p < 0.01, and ***p < 0.001

To further confirm the in vitro findings, we then verified the biological role of circMAPK1 in affecting tumorigenicity in vivo. SGC7901-sh and MGC803-sh cell lines with stable knockdown of circMAPK1 were constructed. These two stable cell lines were inoculated subcutaneously into nude mice, and the status of subcutaneous tumors was observed every week. The silencing of circMAPK1 dramatically promoted tumor growth. In contrast, MKN45 and BGC823 cell lines overexpressing circMAPK1 significantly inhibited the growth of subcutaneous tumors (Fig. 3a). Next, we monitored xenograft tumor proliferation by staining for Ki67, a marker of proliferating cells. The tumor tissues with stable knockdown of circMAPK1 displayed stronger Ki67 staining, while the tumor tissues generated from circMAPK1-overexpressing cells exhibited weaker Ki67 staining (Fig. 3b and c).

To investigate the metastatic potential of circMAPK1 in vivo, the abovementioned cell lines were stably transfected with a luciferase plasmid and then injected into the tail veins of nude mice. Five weeks after the injection, the mice were assessed using the IVIS Spectrum Imaging System, and lung tissue was collected after the mice were sacrificed. The results showed that circMAPK1 knockdown effectively increased the number and size of lung metastatic lesions. In contrast, overexpression of circMAPK1 decreased lung metastatic lesions, as shown by bioluminescence imaging (Fig. 3d and e) and H&E staining (Fig. 3f and g).

### CircMAPK1 encodes a 109-amino acid (aa) novel protein, MAPK1–109aa

**Fig. 4:**
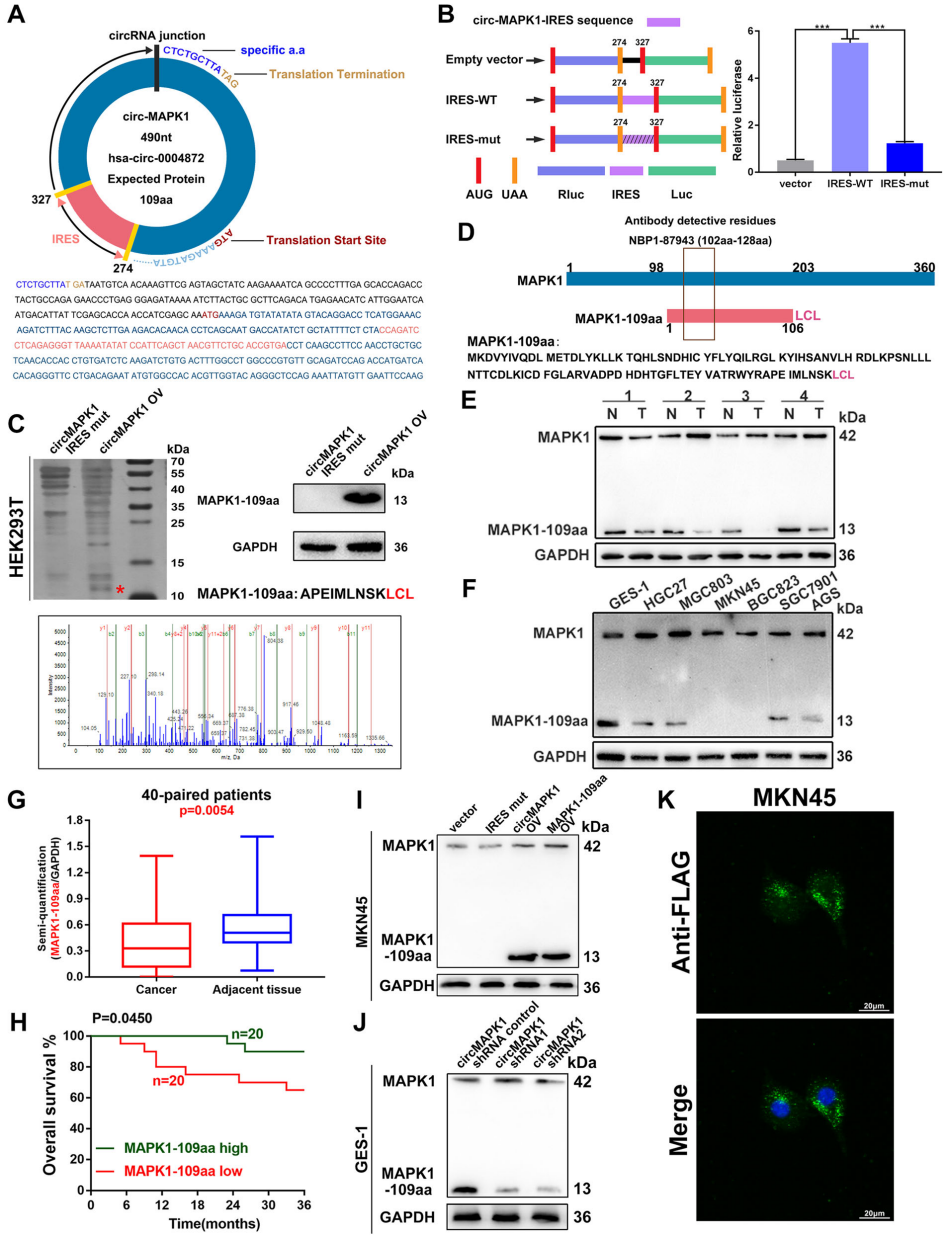
Verification of the coding ability of circMAPK1. **a** Upper panel, the putative open reading frame (ORF) in circMAPK1. Lower panel, the sequences of putative ORF are shown. **b** Left panel, the wild or mutate type of IRES was cloned between the Rluc and Luc reporter genes with independent start (AUG) and stop (UGA) codons. Right panel, the relative luciferase activity was tested. **c** Illustration of MAPK1 sequence and MAPK1–109aa sequence. The antibody used in the study recognized both proteins. **d** The total protein of HEK293T cells transfected with circMAPK1 and IRES-mut plasmids was separated by SDS-PAGE. Immunoblotting confirmed the overexpression of MAPK1–109aa. Cut the difference gel band between 10 kDa and 15 kDa and perform LC-MS/MS analysis. The identified MAPK1–109aa amino acids are shown in red. **e** MAPK1 and MAPK1–109aa expression were detected in GC tissues and its paired normal tissues. **f** MAPK1 and MAPK1–109aa expression were detected in cultured GC cell lines. **g** Semi-quantitative analysis of MAPK1–109aa in GC tissues. **h** Overall survival analysis based on MAPK1–109aa expression in 40 GC patients. **i** The expression of MAPK1 and MAPK1–109aa was detected in MKN45 cells transfected with empty vector, IRES mutated circMAPK1 vector, circMAPK1 vector and MAPK1–109aa vector. **j** The expression of MAPK1 and MAPK1–109aa was detected in GES-1 cells transfected with control shRNA or circMAPK1 shRNAs. **k** Flag tagged MAPK1–109aa was transfected into MKN45 cells. Immunofluorescence staining using anti-Flag was performed to show the MAPK1–109aa cellular localization. Scale bars: 20 μM. Graph represents mean ± SD; *p < 0.05, **p < 0.01, and ***p < 0.001

According to the prediction results of the online database circRNADb, an open reading frame (ORF) with the initiation codon ATG and an IRES at 274-327 nt were contained in the sequence of circMAPK1. This observation suggests that circMAPK1 has the potential to encode a protein of 109 aa, which was termed MAPK1–109aa in this study (Fig. 4a). To verify the activity of the predicted IRES in circMAPK1, a dual-luciferase assay was performed, which showed that the luciferase activity of the wild-type IRES reporter was significantly increased compared with that of the mutated IRES reporter (Fig. 4b). Driven by this IRES, the 109 aa product shares the same sequence at aa 98–203 with wild-type MAPK1, but it contains the special C-terminal sequence Leu-Cys-Leu.

To further confirm the existence of MAPK1–109aa, we transferred the circMAPK1 IRES mutant plasmid and circMAPK1 overexpression plasmid into HEK293T cells. The results of silver staining showed that there was an obvious protein band at a molecular weight of 13 kDa, which was exactly the predicted size of MAPK-109aa. The 13 kDa protein band was excised and subjected to LC-MS/MS. MS detected the sequence of AMEIMLNSKLCL, which was consistent with the amino acids sequence of MAPK1–109aa containing the specific C-terminal sequence LCL (Fig. 4c).

To detect this polypeptide product, we used an antibody that recognized the middle part (102–128 aa) of MAPK1, which means that this antibody can also recognize MAPK1–109aa (Fig. 4d). Western Blot was conducted to detect the levels of MAPK1–109aa and MAPK1 in 40 paired gastric cancer tissues (Fig. 4e and Additional file 3: Fig. S3A to S3I) and cell lines (Fig. 4f). By semi-quantitative analysis, reduced MAPK1–109aa levels were detected in gastric cancer tissues compared with those in normal gastric tissues (Fig. 4g), while MAPK1 expression was increased (Additional file 3: Fig. S3J). Further survival curve showed that GC patients with higher expression of MAPK1–109aa had significantly longer overall survival than those with the lower MAPK1–109aa expression by Kaplan–Meier survival analysis (Fig. 4h). In addition, we also analyzed the correlation between MAPK1, circMAPK1 and MAPK1–109aa in 40 gastric cancer tissues. The MAPK1–109aa expression level showed a clear positive correlation with circMAPK1 (Additional file 3: Fig. S3K), while MAPK1 expression showed no significant correlation with circMAPK1 or MAPK1–109aa (Additional file 3: Fig. S3L and S3M). The content of MAPK1–109aa in a series of GC cell lines was also significantly lower than that in the normal gastric mucosal cell line GES-1. This result was consistent with the RNA expression of circMAPK1 in tissues and cell lines. In MKN45 cells, which have lower levels of endogenous circMAPK1 and almost no MAPK1–109aa expression, the transfection of both circMAPK1 and MAPK1–109aa plasmids produced the predicted MAPK1–109aa band, while overexpression of the circMAPK1 IRES mutant plasmid did not (Fig. 4i). Conversely, in GES-1 cells, which have higher endogenous expression of circMAPK1 and MAPK1–109aa, two shRNAs specifically targeting the junction of circMAPK1 were found to significantly reduce the expression of MAPK1–109aa (Fig. 4j). Immunofluorescence using an anti-Flag antibody confirmed that MAPK1–109aa-Flag was located in the cytoplasm of MKN45 cells overexpressed with MAPK1–109aa-Flag plasmid, as shown in Fig. 4k.

Overall, circMAPK1 encodes a novel MAPK1–109aa protein in GC cells.

### MAPK1–109aa exerts an inhibitory effect on gastric cancer

**Fig. 5:**
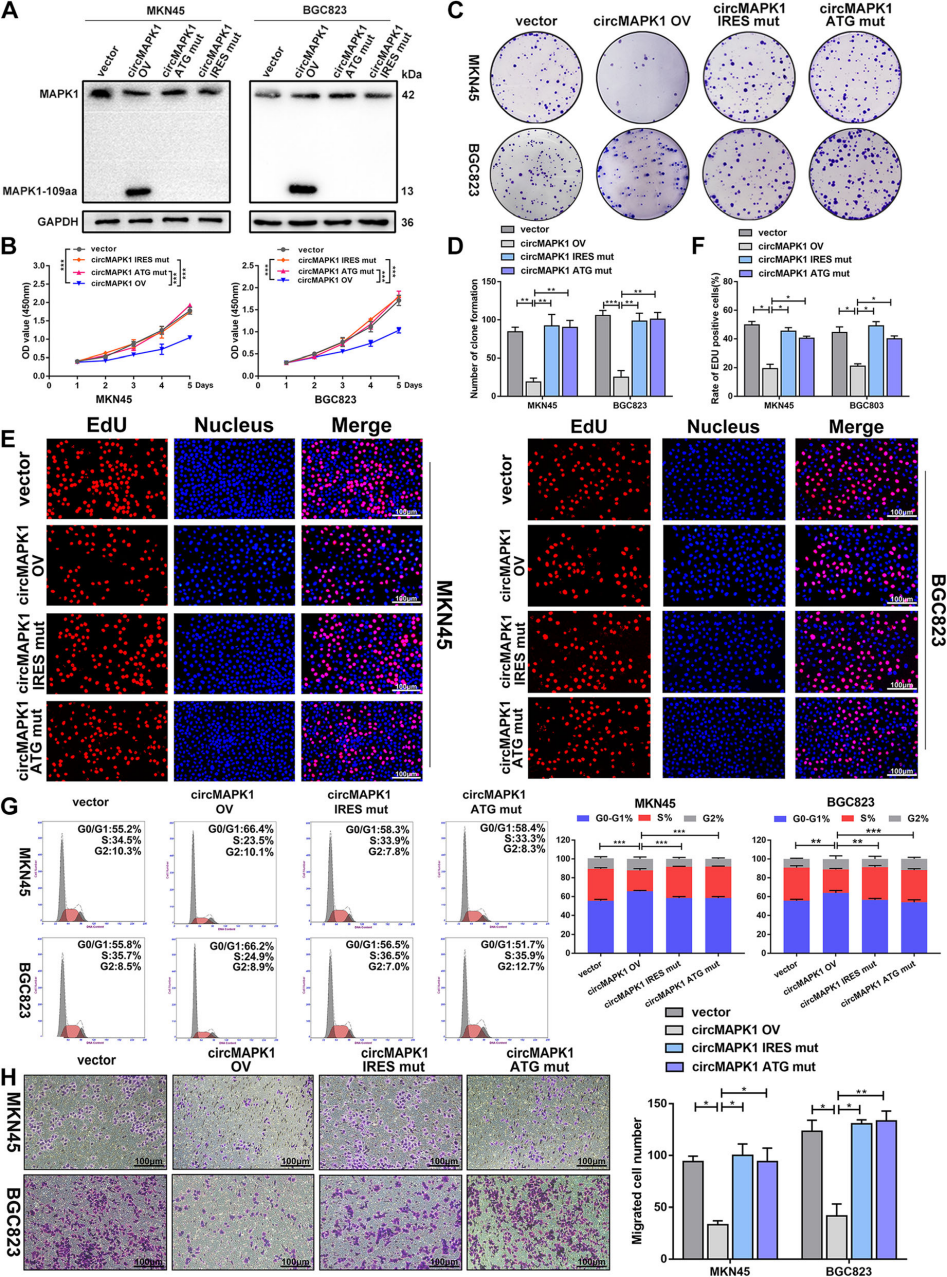
MAPK1–109aa, not circMAPK1, exerts the biological function in GC cells. **a** MKN45 and BGC823 cells were transfected with empty vector, circMAPK1 vector, ATG mutated circMAPK1 vector and IRES mutated circMAPK1 vector. MAPK1–109aa was determined by immunoblot. **b** CCK-8 assay was performed to detect proliferation ability of cells mentioned above. **c** Colony formation assay was performed to detect proliferation ability of cells mentioned above. **d** The number of cells is counted with image J. **e** EdU assay was performed to detect proliferation ability of cells mentioned above. Scale bar: 100 μm. **f** The number of cells is counted with image J. **g** The effect of four plasmids mentioned above on modulating cell cycle progression was evaluated by flow cytometry assay. **h** The effect of four plasmids mentioned above on cell migration was examined by Transwell assay. Scale bar: 100 μm. Graph represents mean ± SD; *p < 0.05, **p < 0.01, and ***p < 0.001

To further investigate the biological function of MAPK1–109aa, we transfected a circMAPK1 ATG mutant plasmid (the start codon of circMAPK1 was mutated), circMAPK1 IRES mutant plasmid and circMAPK1 overexpression plasmid into MKN45 and BGC823 cells. As expected, when the tumor cells were transfected with the ATG mutant plasmid and the IRES mutant plasmid, the MAPK1–109aa 13-kDa band encoded by circMAPK1 did not appear (Fig. 5a). The CCK8 assay (Fig. 5b), colony formation assay (Fig. 5c and d) and EdU assay (Fig. 5e and f) showed that overexpression of circMAPK1 obviously inhibited the proliferation ability. The number of GC cells in S phase was also significantly reduced, as demonstrated by flow cytometry (Fig. 5g). However, when the ATG or IRES sequence of circMAPK1 was mutated, the proliferation ability of MKN45 and BGC823 cells did not change noticeably. Similarly, the migration ability of tumor cells showed analogous results, as evidenced by the Transwell assay (Fig. 5h) and wound healing assay (Additional file 3: Fig. S4A and S4B). Collectively, these results indicate that circMAPK1 could not suppress the malignant phenotype of GC cells without its encoded protein MAPK1–109aa.

**Fig. 6:**
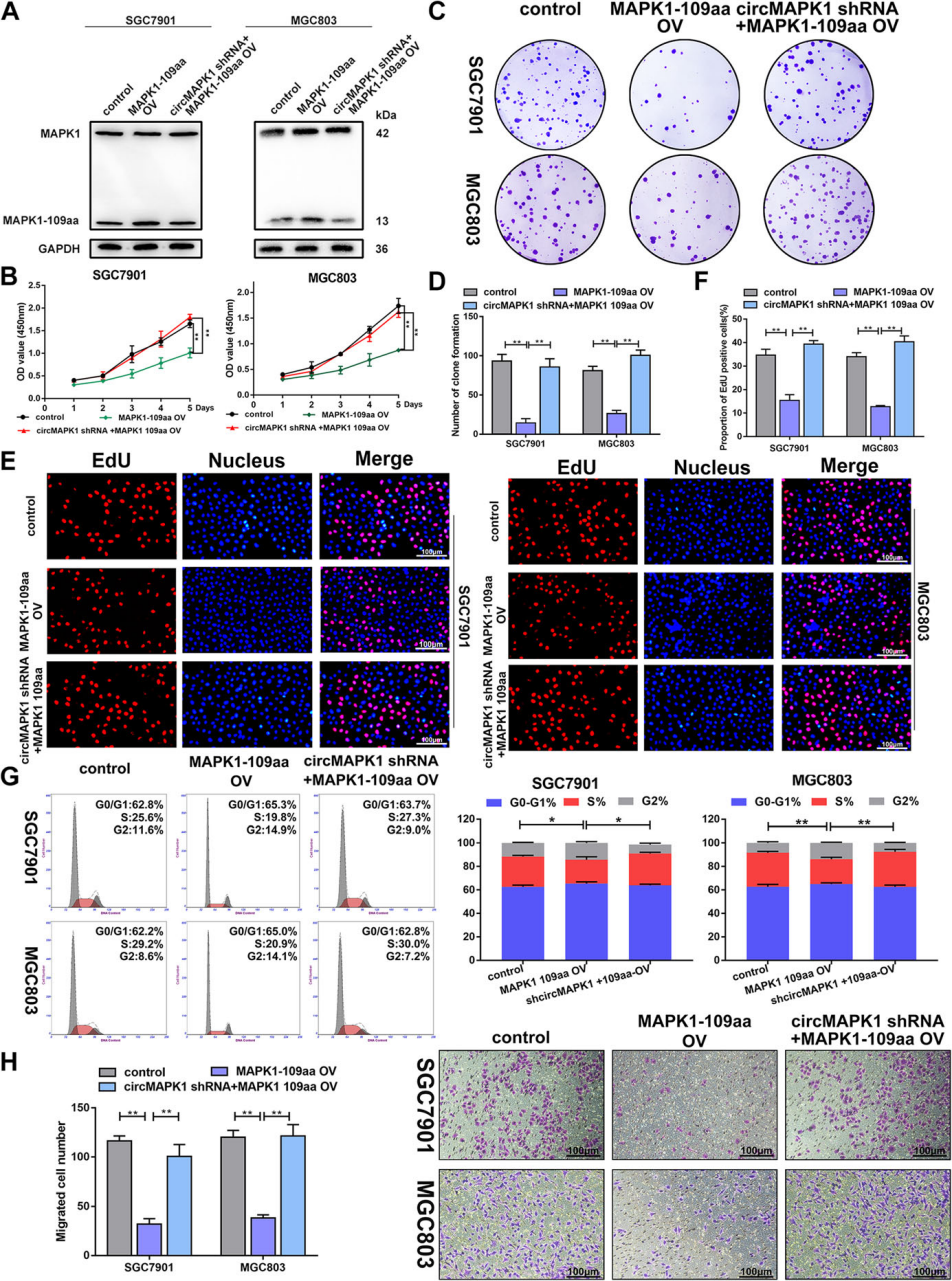
Tumor suppressive functions of MAPK1–109aa in GC cell lines. **a** SGC7901 and MGC803 cells were transfected with circMAPK1 shRNA or circMAPK1 shRNA plus linearized MAPK1–109aa overexpression plasmid. MAPK1–109aa expression was decided by immunoblot. **b** CCK-8 assay was performed to detect proliferation ability of cells mentioned above. **c** Colony formation assay was performed to detect proliferation ability of cells mentioned above. **d** The number of cells is counted with image J. **e** EdU assay was performed to detect proliferation ability of cells mentioned above. Scale bar: 100 μm. **f** The number of cells is counted with image J. **g** The effect of plasmids mentioned above on modulating cell cycle progression was evaluated by flow cytometry assay. **h** The effect of plasmids mentioned above on cell migration was examined by Transwell assay. Scale bar: 100 μm. Graph represents mean ± SD; *p < 0.05, **p < 0.01, and ***p < 0.001

Subsequently, to further verify the function of MAPK1–109aa, we restored MAPK1–109aa expression in SGC7901 and MGC803 cells with or without the stable knockdown of circMAPK1, which were verified by Western blot (Fig. 6a). After the MAPK1–109aa plasmid was transfected into the SGC7901 and MGC803 cell lines, the cell proliferation ability (Fig. 6b-g) and migration ability (Fig. 6h and Additional file 3: Fig. S4C and S4D) were effectively inhibited. In the cell lines transfected with shcirMAPK1, restored MAPK1–109aa expression also reversed the malignant phenotypes induced by the stable knockdown of circMAPK1.

In summary, the above results demonstrated that the inhibitory effect of circMAPK1 on the malignant biological behavior of GC cells was dependent on its encoded protein MAPK1–109aa.

### MAPK1–109aa inhibits the phosphorylation of MAPK1 by competitively binding to MEK1

**Fig. 7:**
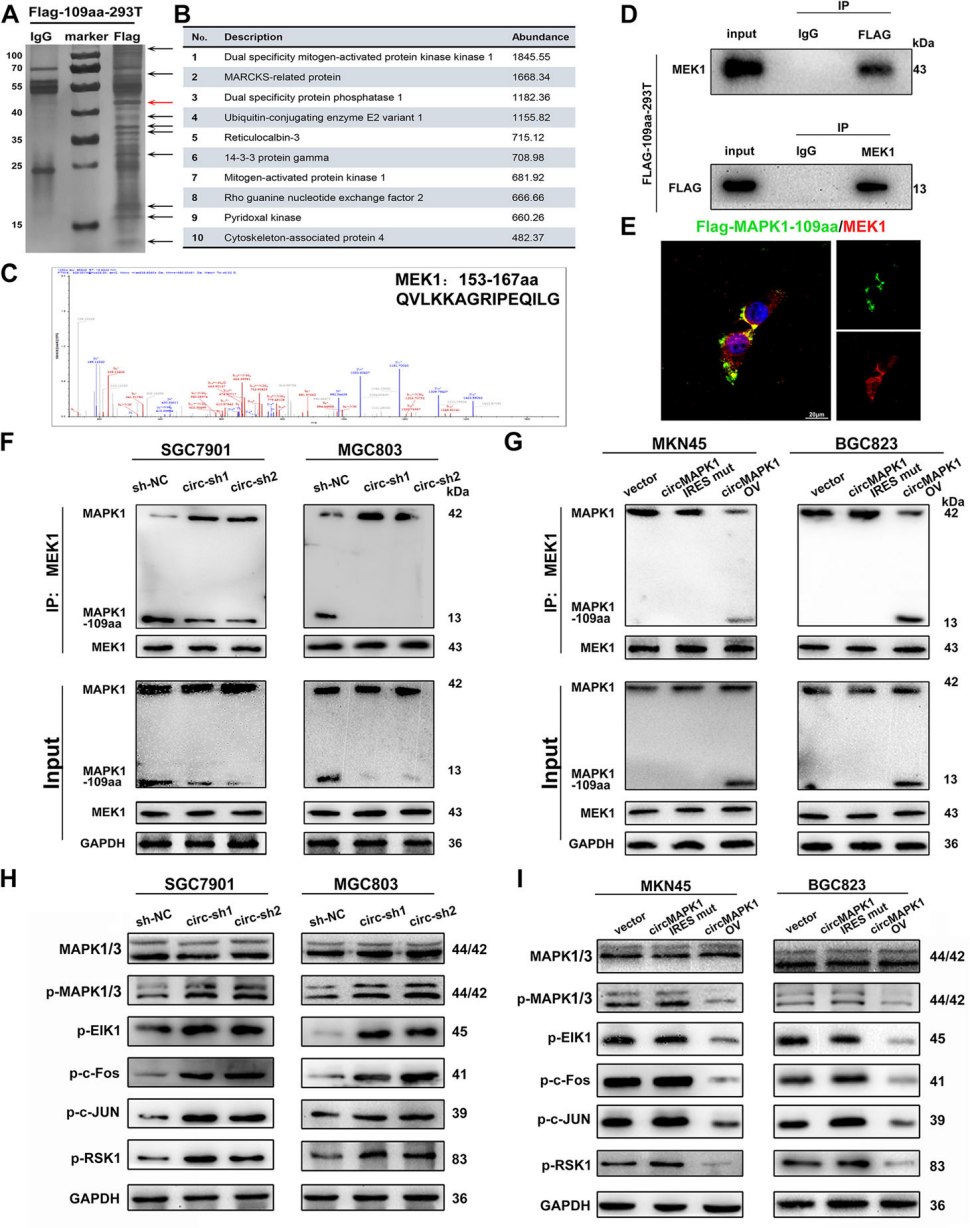
MAPK1–109aa inhibits phosphorylation of MAPK1 by competitively binding to MEK1. **a** Total protein from Flag-MAPK1–109aa plasmid-transfected HEK293T cells were separated via SDS-PAGE. Arrows show different bands between IgG and Flag. **b** List of the top ten differentially expressed proteins identified by mass spectrometry. **c** MEK1 was identified by LC/LC-MS in MAPK1–109aa protein complex. **d** Mutual interaction of MEK and Flag-MAPK1–109 aa were determined by IP. **e** Flag-tagged MAPK1–109aa was transfected into HEK293T cells and immunofluorescence was performed using anti-Flag and anti-MEK1 antibody. Scale bar, 20 μm. **f** In SGC7901 and MGC803 cell lines with stable knockout of circMAPK1, the expression level of MAPK1–109aa bound to MEK1 decreased. **g** The expression level of MAPK1–109aa bound to MEK1 increased in MKN45 and BGC823 cell lines transferred into circMAPK1 vector. **h** In SGC7901 and MGC803 cell lines with stable knockout of circMAPK1, the expression level of downstream cytokines p-ElK1, p-c-Fos, p-c-JUN, and p-RSK1 in the MAPK pathway increased significantly. **i** The expression level of downstream cytokines p-ElK1, p-c-Fos, p-c-JUN, and p-RSK1 in the MAPK pathway decreased in MKN45 and BGC823 cell lines transferred into circMAPK1 vector

To investigate the molecular mechanism by which MAPK1–109aa inhibits GC, we transfected the Flag-labeled MAPK1–109aa plasmid into HEK293T cells. The potential interacting protein was pulled down in the Flag-tagged MAPK1–109aa immunoprecipitation complex (Fig. 7a). Protein MS analysis was used to identify the differentially expressed proteins. In the ranking list of recognized proteins, the protein with the highest abundance was MEK1, suggesting that MEK1 was a potential interacting protein of MAPK1–109aa (Fig. 7b and c). Further, Flag-tagged MAPK1–109aa could mutually interact with MEK1, confirming the direct interaction between MAPK1–109aa and MEK1 (Fig. 7d). Subsequent immunofluorescence staining further showed the colocalization between MAPK1–109aa and MEK1 (Fig. 7e).

The MAPK pathway relies on the transmission of extracellular signals to intracellular signals and is activated by a variety of transmembrane receptors. The activated upstream kinase MEK1 phosphorylates its only substrate, MAPK1, at the phosphorylation sites Thr185 and Tyr187. Phosphorylated MAPK1 subsequently activates a series of transcription factors and regulatory molecules, thereby participating in various physiological activities of cells. Since MAPK1–109aa shares the same sequences at 98–203 amino acid with MAPK1, which includes the phosphorylation sites of MAPK1, we hypothesized that MAPK1–109aa may compete with MAPK1 for binding to MEK1.

Immunoprecipitation with a MEK1 antibody was performed to explore the potential regulatory effect of MAPK1–109aa on the MAPK1 pathway. We found that in SGC7901 and MGC803 cells with the stable knockdown of circMAPK1, MAPK1–109aa binding to MEK1 clearly decreased, while MAPK1 binding to MEK1 increased compared with that in the control (Fig. 7f). In MKN45 and BGC823 cells, MAPK1–109aa was not translated when the IRES was mutated. As a result, the binding of MAPK1 to MEK1 did not change significantly. However, after overexpression of circMAPK1, the binding of MAPK1 to MEK1 significantly decreased, while the binding of MAPK1–109aa to MEK1 notably increased (Fig. 7g). Therefore, the above results indicated that MAPK1–109aa competed with MAPK1 for binding to MEK1.

Subsequent Western blot also confirmed that in cell lines overexpressing circMAPK1, MAPK1–109aa was significantly increased, while phosphorylated MAPK1/2 was reduced, which was the opposite of the results in circMAPK1-knockdown cells (Fig. 7h). Furthermore, MAPK1 downstream effectors, such as p-ELK1, p-c-Fos, p-c-JUN, and p-RSK, were also significantly inhibited by overexpression of circMAPK1 (Fig. 7i). These findings indicate that MAPK1–109aa encoded by circMAPK1 acted as a tumor suppressor by inhibiting the MAPK1 signaling pathway.

According to the MS assay, MRP, DUSP1, UBE2V1 and RCN-3 are some other proteins of highly binding possibilities with MAPK1–109aa since they have high abundance in mass spectrometry. We subsequently construct transient knock-down systems of these proteins in gastric cancer cell lines. Western blot experiments showed that the phosphorylation of MAPK was not significantly changed when we blocked expression of MRP, UBE2V1 and RCN-3, and the expression of downstream effect factors was not significantly changed either (Additional file 3: Fig. S5A and S5B). However, the phosphorylation of MAPK increased slightly when DUSP1 was interfered, which indicated that DUSP1 could downregulate the phosphorylation of MAPK. Subsequent co-Immunoprecipitation was performed to confirm whether the direct interaction exist between DUSP1 and MAPK1–109aa. The result showed that Flag-tagged MAPK1–109aa could not interact with DUSP1, which indicated MAPK1–109aa could affect the MAPK pathway independent of DUSP1 (Additional file 3: Fig. S5C).

**Fig. 8:**
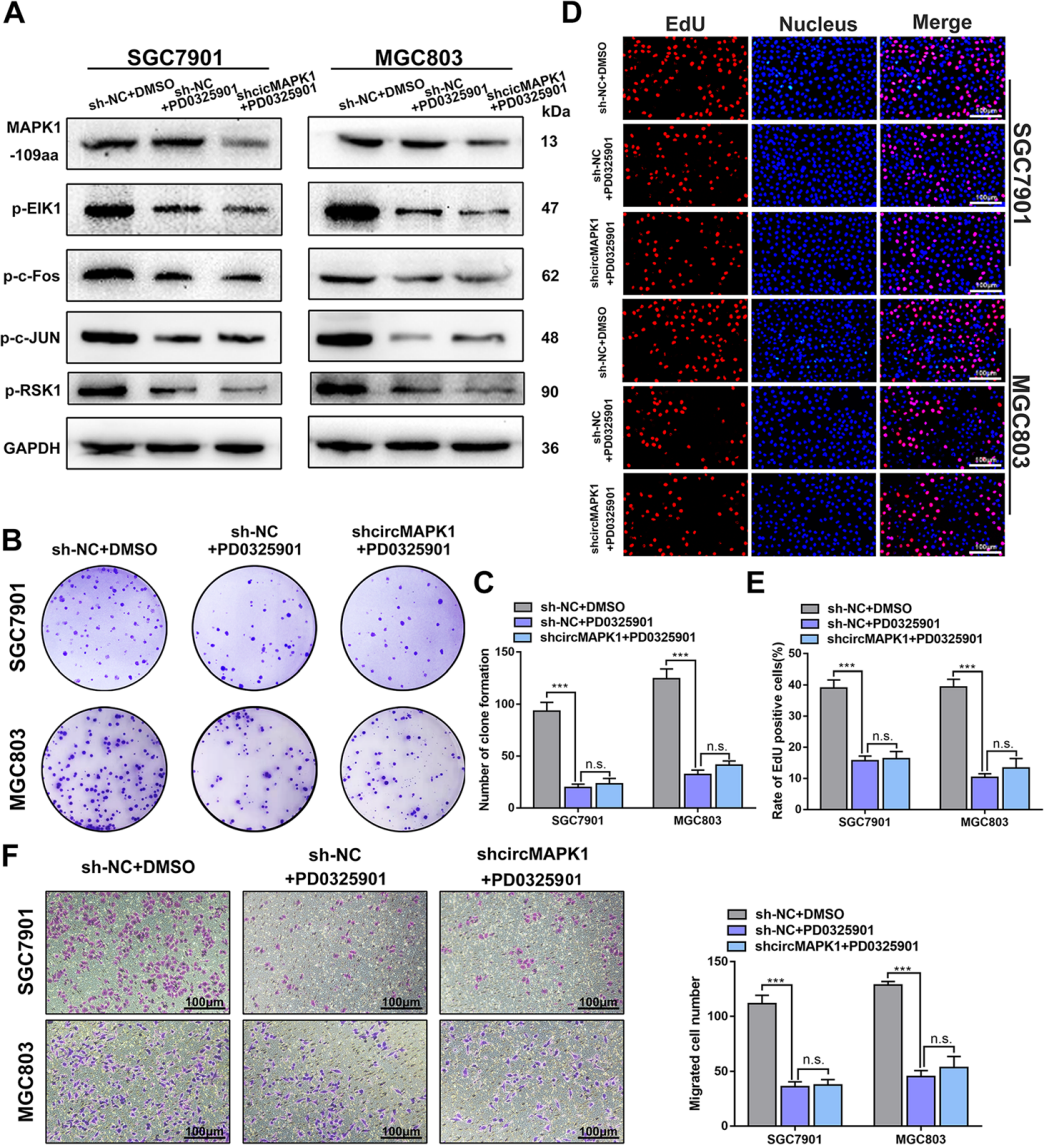
MAPK1–109aa play a tumor suppressor role merely through the MAPK pathway. **a** After adding shcircMAPK1 NC, MEK1 inhibitor (PD0325901), shcircMAPK1 plus PD0325901 to 7901 and 803 cell lines, the expression levels of MAPK1–109aa and downstream cytokines in the MAPK pathway. **b** Colony formation assay was performed to detect proliferation ability of cells mentioned above. **C** The number of cells is counted with image J. **d** EdU assay was performed to detect proliferation ability of cells mentioned above. Scale bar: 100 μm. **e** The number of cells is counted with image J. **f** Left panel: The effect of addition mentioned above on cell migration was examined by Transwell assay. Scale bar: 100 μm. Right panel: Quantitative of the cells by image J. Graph represents mean ± SD; *p < 0.05, **p < 0.01, and ***p < 0.001

The MAPK cascade is one of the most important oncogenic drivers of human cancers, and the blockade of this signaling pathway by targeted inhibitors is an important antitumor strategy. Therefore, we treated SGC7901 and MGC803 cells with a MAPK pathway inhibitor, PD0325901. This inhibitor has been proven to effectively inhibit the phosphorylation of MAPK1 and induce apoptosis of tumor cells. Western blot experiments showed that the addition of PD0325901 significantly reduced the activation of downstream factors of the MAPK1 pathway. However, the expression level of MAPK1–109aa did not changed significantly after adding PD0325901 (Fig. 8a). As expected, the proliferation and migration abilities of cells treated with PD0325901 were significantly suppressed assessed by colony formation (Fig. 8b and c), EdU (Fig. 8d and e) and Transwell assay (Fig. 8f). Further, in the presence of the inhibitor, transfection of shcircRNA into SGC7901 and MGC803 cells was intend to partially enhance the activity of the MAPK pathway. However, circMAPK1 repression showed no obvious cancer-promoting effects when cells were co-treated with PD0325901 (Fig. 8b-f).

Moreover, C16-PAF, a MAPK1 pathway stimulator, was added to the MKN45 cells. The activation of the MAPK1 pathway downstream factors was verified by Western blot (Additional file 3: Fig. S6A) and the proliferation and invasion abilities of GC cells were greatly increased (Additional file 3: Fig. S6B to S6G). Subsequent additional transfection with circMAPK1 overexpressing plasmid antagonized the promoting effects of C16-PAF in GC cells.

Taken together, these results verified that MAPK1–109aa inhibits the phosphorylation of MAPK1 by competitively interacting with MEK1, exerts the tumor suppressive effect by suppressing MAPK pathway.

## Discussion

MAPK cascades are key signaling pathways involved in the regulation of normal cell proliferation, survival and differentiation [27]. The aberrant overactivation of MAPK cascades contributes to cancers and other human diseases [28–31]. Recently, the application of compounds targeting components of the MAPK pathway (such as RAF and MEK inhibitors) has been proven to substantially improve the clinical outcome of metastatic melanoma and can also show surprising clinical effects in other tumors [32]. However, the response to the drugs varies greatly, and the clinical efficacy of these drugs is mainly limited by the development of drug resistance [33]. Acquired drug resistance often occurs within 1 year of therapy and has become an obstacle to the long-term survival of clinical patients [34]. It has been proposed that the combined use of selective MAPK1/2 inhibitors can expand the treatment scope to tumors driven by the MAPK pathway [35]. Ulixertinib, MK-8353 and GDC-0994 are orally effective and specific MAPK inhibitors that are being applied in early clinical trials for the treatment of numerous advanced/metastatic solid tumors [36]. Additional work will be required to determine whether MAPK inhibitors are clinically successful, and whether they can prevent the development of resistance through the MAPK pathway is also unknown. Therefore, more innovative methods are needed to target this pathway.

As a new type of RNA, circRNA has a unique circular structure that is derived from the back-splicing biogenesis process [37]. Recently, with the rapid development of high-throughput sequencing technology and bioinformatics analysis, a great number of circRNAs have been found in mammalian cells [38]. Abnormal expression of circRNAs has been reported in numerous human diseases, including cancer, neurologic diseases, endocrine diseases, and cardiovascular diseases [39]. Owing to their abundance, high stability, tissue-specific expression pattern, and wide distribution in various body fluids, circRNAs have great potential as biomarkers of human diseases. Here, we focused on circRNAs arisen from the MAPK pathway. We identified the circRNA hsa_circ_0004872, termed circMAPK1 in this article, through the analysis and screening of the circBase database. The expression of circMAPK1 was relatively downregulated in GC. Further survival analysis also showed that higher expression of circMAPK1 was associated with longer survival time of GC patients, which indicated that circMAPK1 had a potential role in the occurrence and development of GC and that it might be a therapeutic target.

Since unlimited cell proliferation, migration and metastasis are the hallmarks of malignant tumors, we next explored the role of circMAPK1 in the progression of GC through gain- and loss-of-function experiments. A series of in vitro and in vivo functional experiments showed that circMAPK1 inhibited the proliferation, migration and metastasis of GC. We then focused our research on the mechanism by which circMAPK1 exerts its tumor suppressor function.

In recent years, research on circRNAs related to GC has received increasing attention [40]. A considerable number of articles have confirmed that circRNAs can affect the malignant biological behavior of GC by acting as miRNA sponges or interacting with related proteins [41]. For example, circHuR inhibits the expression of HuR and the progression of GC by suppressing CNBP transactivation [42]. CircHECTD1 promotes glutamine breakdown by targeting miR-1256 and activating β-catenin/c-Myc signaling to promote the progression of GC [43]. A small number of studies have found that circRNAs can affect cell proliferation, differentiation, metastasis and other processes by encoding proteins [12], challenging their traditional role as noncoding RNA molecules. Our research showed that circMAPK1 encodes a 109-aa-long protein (MAPK1–109aa). Because the aa sequence of the ORF includes the cyclization site of circMAPK1, MAPK1–109aa shares most of its aa sequence with MAPK1, except for the unique aa sequence LCL in the C-terminus. Subsequent functional experiments also confirmed that MAPK1–109aa mediates the inhibitory effect of circMAPK1.

To explore the mechanism of action of MAPK1–109aa, coimmunoprecipitation followed by MS was conducted using Flag-tagged MAPK1–109aa as bait. Among the identified precipitated proteins, MAPKK1 (MEK1) was the most abundant, which prompted us to further investigate the regulatory mechanism between MAPK1–109aa and MEK1. MEK1 is a bispecific protein kinase belonging to the MAP kinase kinase (MKK) family and an important component of the MAPK signal transduction pathway [44]. MEK1 plays a key role in the regulation of gene expression and cytoplasmic function by transmitting upstream signals to downstream response molecules [45]. MKKs phosphorylate a threonine and a tyrosine in a TXY motif in the MAPK activation loop [46]. In principle, the residues in and around this motif are also involved in the recognition and phosphorylation of the MAPK family by MKKs. That is, MEK1/2 could recognize the sequence FLTEY in MAPK1/2, which may be important for the mutual recognition of MEK1/2 and MAPK1/2. In addition, we found that MAPK1–109aa contains the phosphorylation sites and surrounding sequences of MAPK1, which suggests that MEK1 also has the potential to recognize and phosphorylate MAPK1–109aa. The binding of MAPK1–109aa to MEK1 was confirmed by subsequent immunoprecipitation and Western blot experiments.

Studies focused on the encoding abilities of circRNA have demonstrated that some circRNAs encoding proteins play the inconsistent role with their host gene products. For instance, it has been reported that AKT3-174aa inhibits glioma tumorigenicity by decreasing activated AKT [47]. In contrast, some products encoded by circRNAs are involved in the functions of their host genes. SHPRH-146aa encoded by circSHPPH protects full-length SHPRH from degradation by the ubiquitin proteasome [14]. Here, we found that the product MAPK1–109aa encoded by circMAPK1 interfered with the function of its host gene MAPK1. A coimmunoprecipitation experiment was conducted to confirm that MAPK1–109aa competitively bound to MEK1 and inhibited the phosphorylation of MAPK1, but we did not detect the presence of phosphorylated MAPK1–109aa at a molecular weight of 13 kDa, indicating that MEK1 is unable to phosphorylate this short peptide, even if it contains the sequence of the MAPK1 activation fragment, which was consistent with a previous research [23]. CircRNAs are generated by cells through specific mechanisms, while some circRNAs can be translated into specific short peptides to regulate the function of their parental genes. This may represent an efficient feedback mechanism in cells and deserves further study. Although the sequence of the peptide translated by the circRNA was similar to that of the protein translated by the parent gene, the former has several specific aa differences from the latter. Whether these specific aa have specific functions needs further exploration. Simultaneously, we also proved that MAPK1–109aa inhibited the activation of tumor-promoting factors downstream of the MAPK1 pathway. This further showed that the short peptide MAPK1–109aa encoded by circMAPK1 can negatively regulate the cancer-promoting effect of the MAPK pathway. Besides, what’s more refreshing is that MAPK1–109aa may solve the problem of acquired drug resistance in clinical applications of MAPK pathway inhibitors since it can cooperate with MAPK pathway inhibitors to exert a tumor suppressor effect. Taken together, attributing to the suppressing effects on MAPK pathway activation, the short peptides MAPK1–109aa may be a promising inhibitor, which needs further studies on its clinical application.

## Conclusions

In conclusion, we identified a novel downregulated circRNA derived from MAPK1 in gastric cancer. MAPK1–109aa, encoded by circMAPK1, exerts an anticancer effect by competing with MAPK1 to bind to the upstream kinase MEK1, suppressing the phosphorylation of MAPK1 and the downstream oncogenic factors. Our findings suggested that circMAPK1 may be used as a therapeutic target in GC and it also provides new treatment ideas for diseases caused by the activation of the MAPK pathway.

## Supplementary Information

**Additional file 1.**

**Additional file 2.**

**Additional file 3.**

**Additional file 4.** Materials and Methods.

## Data Availability

The data supporting the conclusions of this article have been given in this article and its additional files.

## Abbreviations

CCK8: Cell counting kit-8
circRNA: Circular RNA
circMAPK1: Circular MAPK1
EdU: 5-Ethynyl-2′-deoxyuridine
FISH: RNA fluorescence in situ hybridization
GC: Gastric cancer
IP: Immunoprecipitation
IRES: Internal ribosomal entry site
MAP K1: Mitogen-activated protein kinase 1
MEK1: Dual specificity mitogen-activated protein kinase kinase 1
ORF: Open reading frame
OS: Overall survival
qRT-PCR: Quantitative real-time PCR
siRNA: Small interfering RNA
