# Supplementary material for: A novel protein encoded by circMAPK1 inhibits progression of gastric cancer by suppressing activation of MAPK signaling
Source: Mol Cancer. 2021 Apr 9;20:66. doi: 10.1186/s12943-021-01358-y (PMC8034133; doi:10.1186/s12943-021-01358-y)
Supplement: Supplementary file 3 — Additional file 3. [file 12943_2021_1358_MOESM3_ESM.docx]

**Supplementary Figure 1**


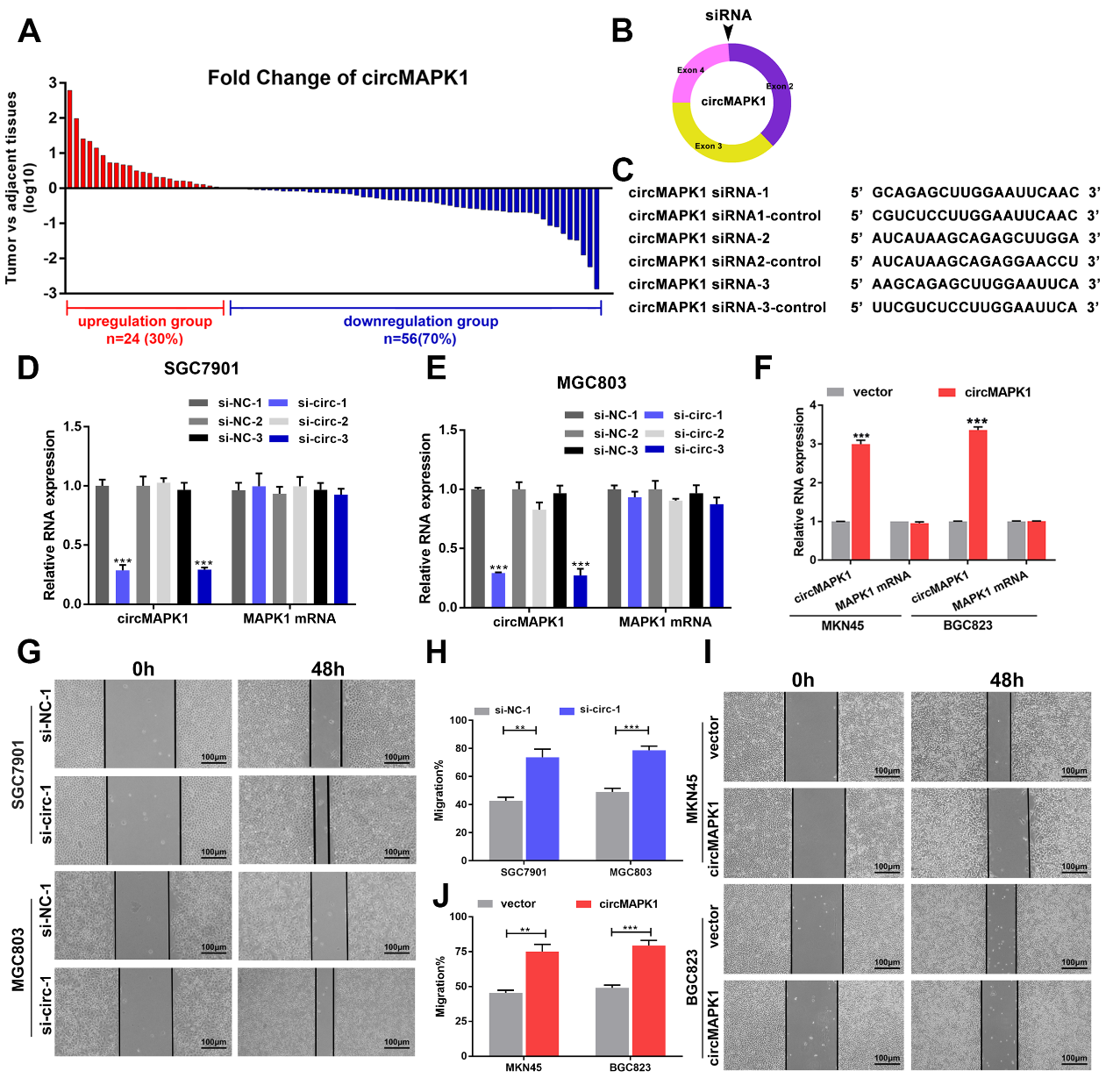


**A** The log10 fold changes of circMAPK1 in each paired GC sample were displayed from high to low.

**B** The diagram of small interfering RNAs (siRNAs) specifically targeting the back-splice junction sequences of circMAPK1.

**C** The sequence of small interfering RNAs (siRNAs) specifically targeting the back-splice junction sequences of circMAPK1.

**D** The efficiencies of siRNA3 in SGC7901 cell line were verified by qRT-PCR.

**E** The efficiencies of siRNA3 in MGC803 cell line were verified by qRT-PCR.

**F** The efficiency of circMAPK1 overexpression vectors was verified by qRT-PCR.

**G&H** Wound healing assay was performed to evaluate migration ability of GC cells after downregulating circMAPK1. Scale bar: 100 μm.

**I&J** Wound healing assay was performed to evaluate migration ability of GC cells after upregulating circMAPK1. Scale bar: 100 μm.

Graph represents mean ± SD; **p* < 0.05, ***p* < 0.01, ****p* < 0.001.

**Supplementary Figure 2**


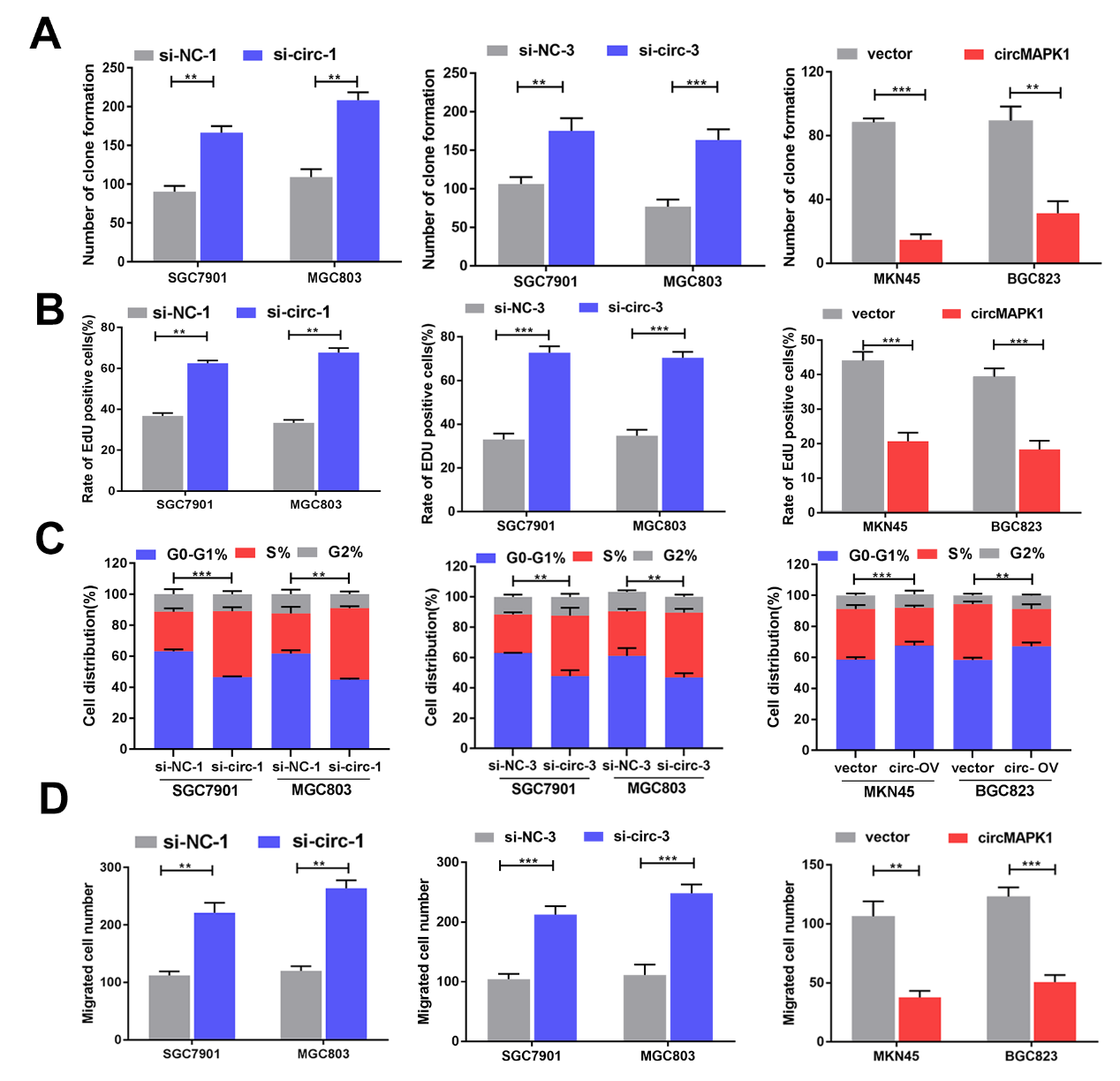


**A** The number of cells detected in colony formation assay is counted with image J.

**B** The rate of EdU positive cells is counted with image J.

**C** Statistics of cell number in different cell cycle phases.

**D** The number of cells detected in transwell assay is counted with image J.

Graph represents mean ± SD; **p* < 0.05, ***p* < 0.01, and ****p* < 0.001.

**Supplementary Figure 3**


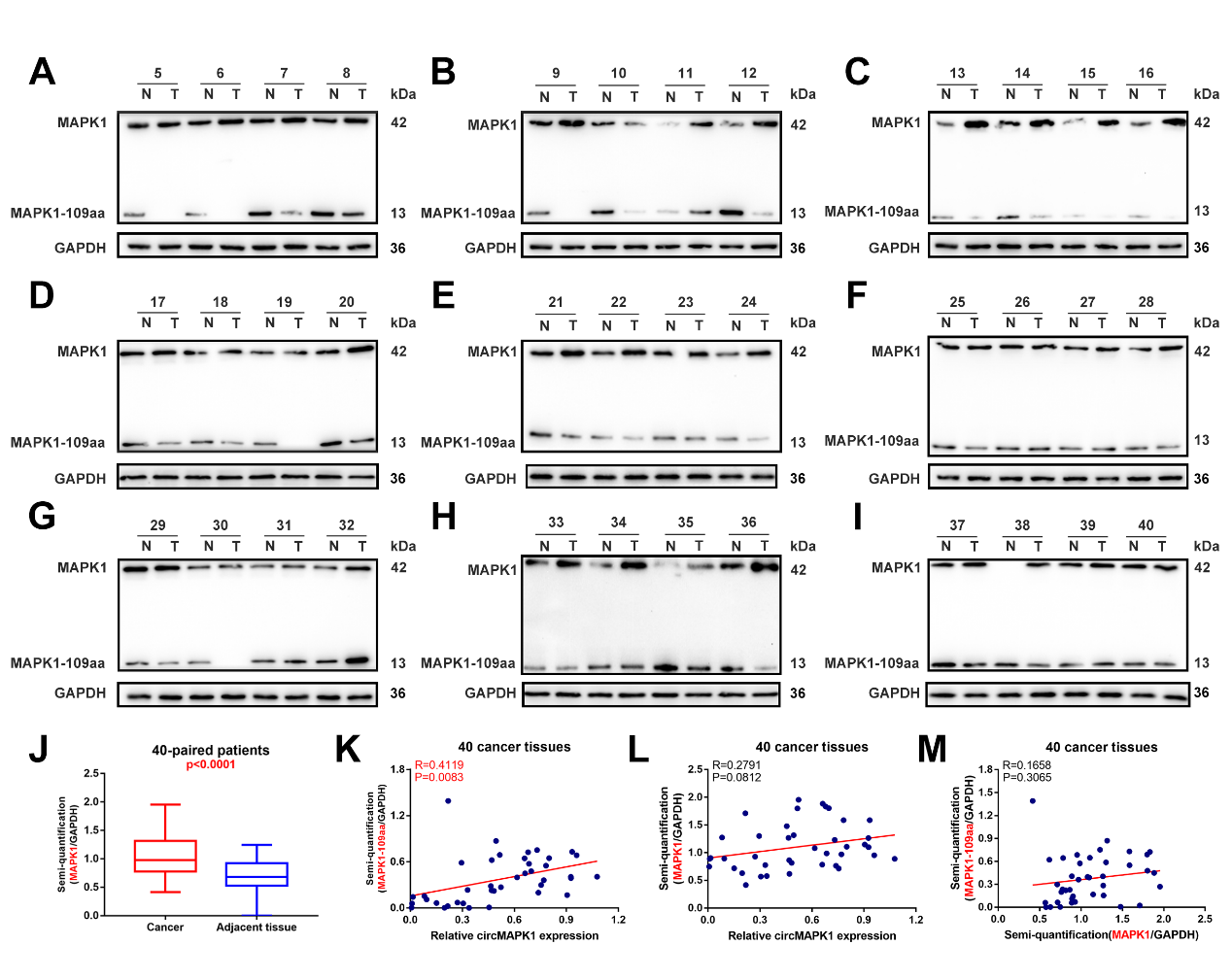


**A-I** MAPK1 and MAPK1-109aa expression were detected in GC tissues and its paired normal tissues.

**J** Semi-quantitative analysis of MAPK1 in GC tissues.

**K** The correlation analysis between circMAPK1 and MAPK1-109aa in GC tissues.

**L** The correlation analysis between circMAPK1 and MAPK1 in GC tissues.

**M** The correlation analysis between MAPK1 and MAPK1-109aa in GC tissues.

**Supplementary Figure 4**


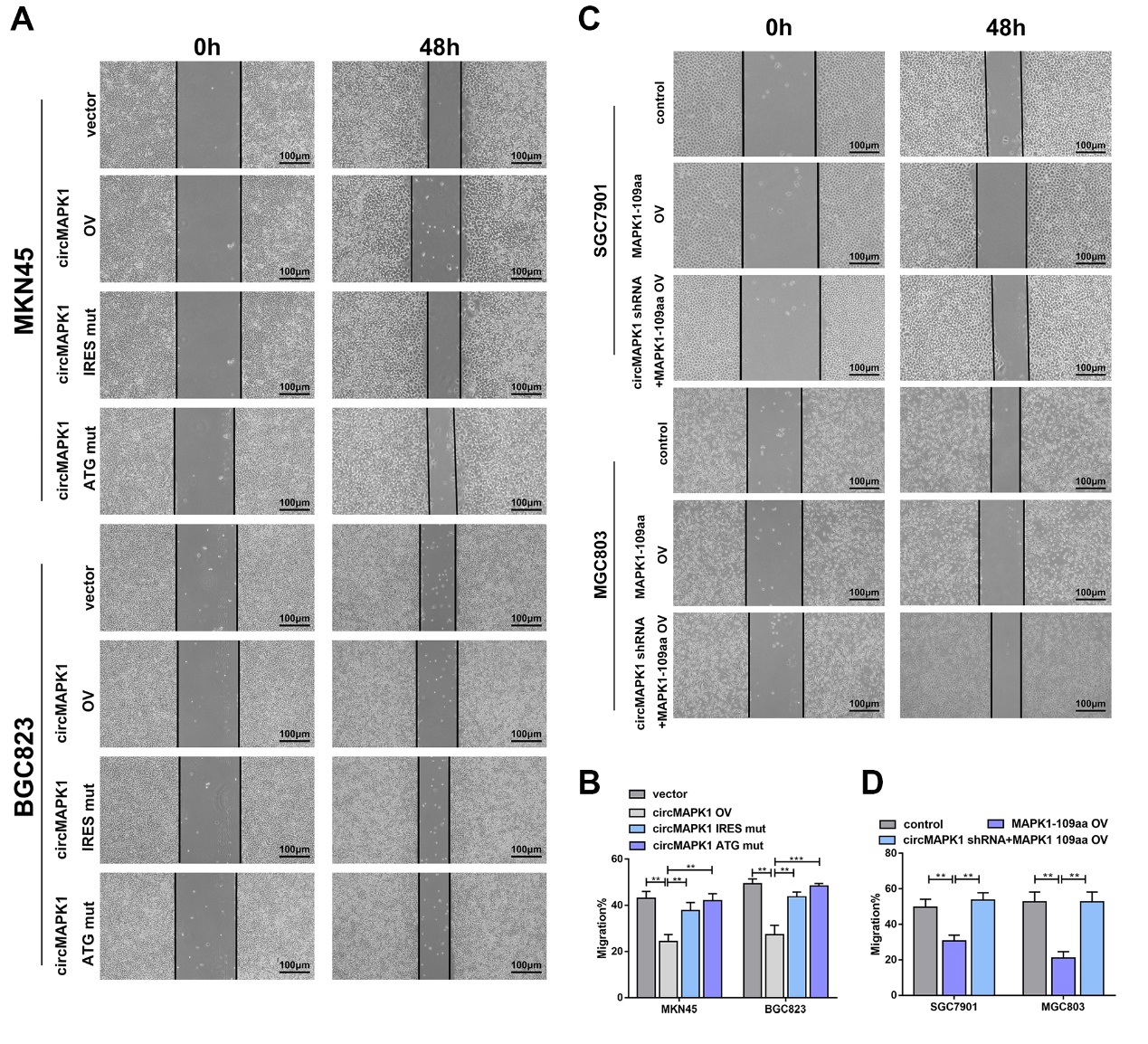


**A&B** Wound healing assay was performed to evaluate migration ability of MKN45 and BGC823 cells transfected with empty vector, circMAPK1 vector, ATG mutated circMAPK1 vector and IRES mutated circMAPK1 vector. Scale bar: 100 μm.

**C&D** Wound healing assay was performed to evaluate migration ability of SGC7901 and MGC803 cells transfected with circMAPK1 shRNA or circMAPK1 shRNA plus linearized MAPK1-109aa overexpression plasmid. Scale bar: 100 μm.

Graph represents mean ± SD; **p* < 0.05, ***p* < 0.01, ****p* < 0.001.

**Supplementary Figure 5**


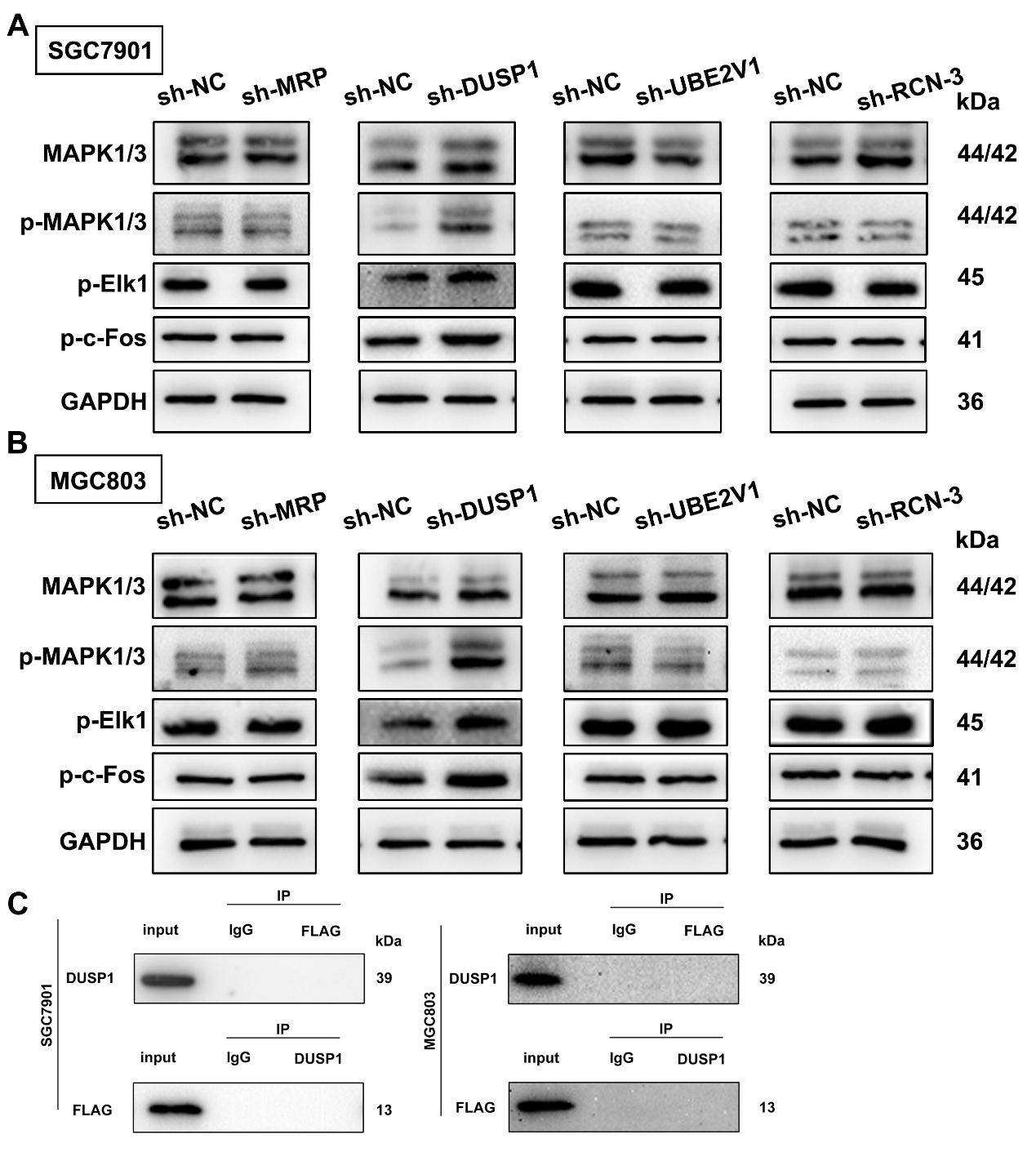


**A** The expression level of p-MAPK and the downstream cytokines p-ElK1, p-c-Fos after interfering MRP, DUSP1, UBE2V1 and RCN-3 in SGC7901.

**B** The expression level of p-MAPK and the downstream cytokines p-ElK1, p-c-Fos after interfering MRP, DUSP1, UBE2V1 and RCN-3 in MGC803.

**C** The interaction of DUSP1 and MAPK1-109 aa was determined by IP.

**Supplementary Figure 6**


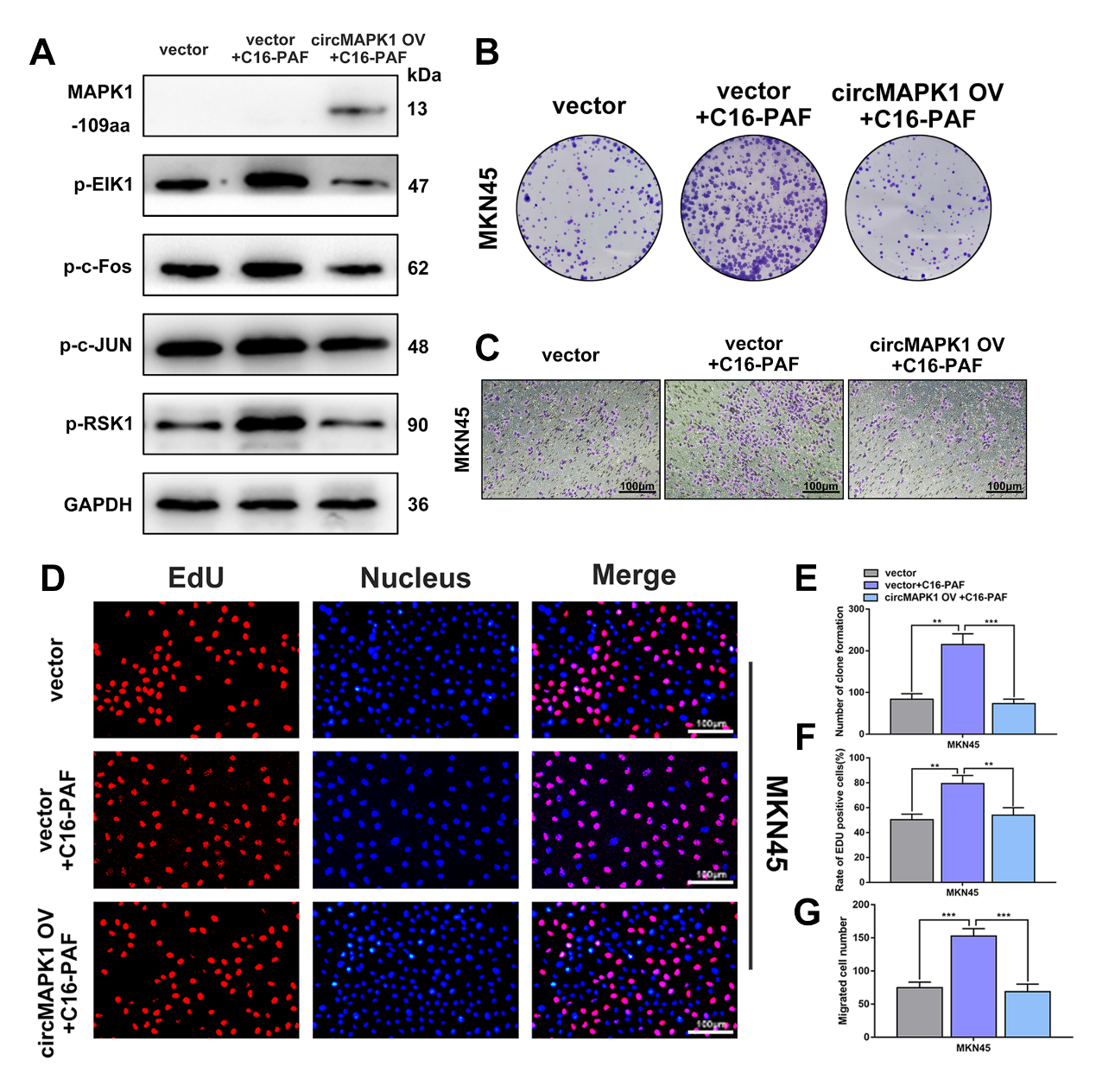


**A** After adding vector, MAPK1 stimulator(C16-PAF), circMAPK1 overexpression plasmid plus MAPK1 stimulator(C16-PAF) to MKN45 cell line, the expression levels of MAPK1-109aa and downstream cytokines in the MAPK pathway

**B** Colony formation assay was performed to detect proliferation ability of cells mentioned above. **E** The number of cells is counted with image J.

**D** EdU assay was performed to detect proliferation ability of cells mentioned above. Scale bar: 100 μm. **F** The number of cells is counted with image J.

**C** The effect of addition mentioned above on cell migration was examined by Transwell assay. Scale bar: 100 µm. **G** Quantitative of the cells by image J

Graph represents mean ± SD; **p* < 0.05, ***p* < 0.01, and ****p* < 0.001.

**Supplementary Figure 7**


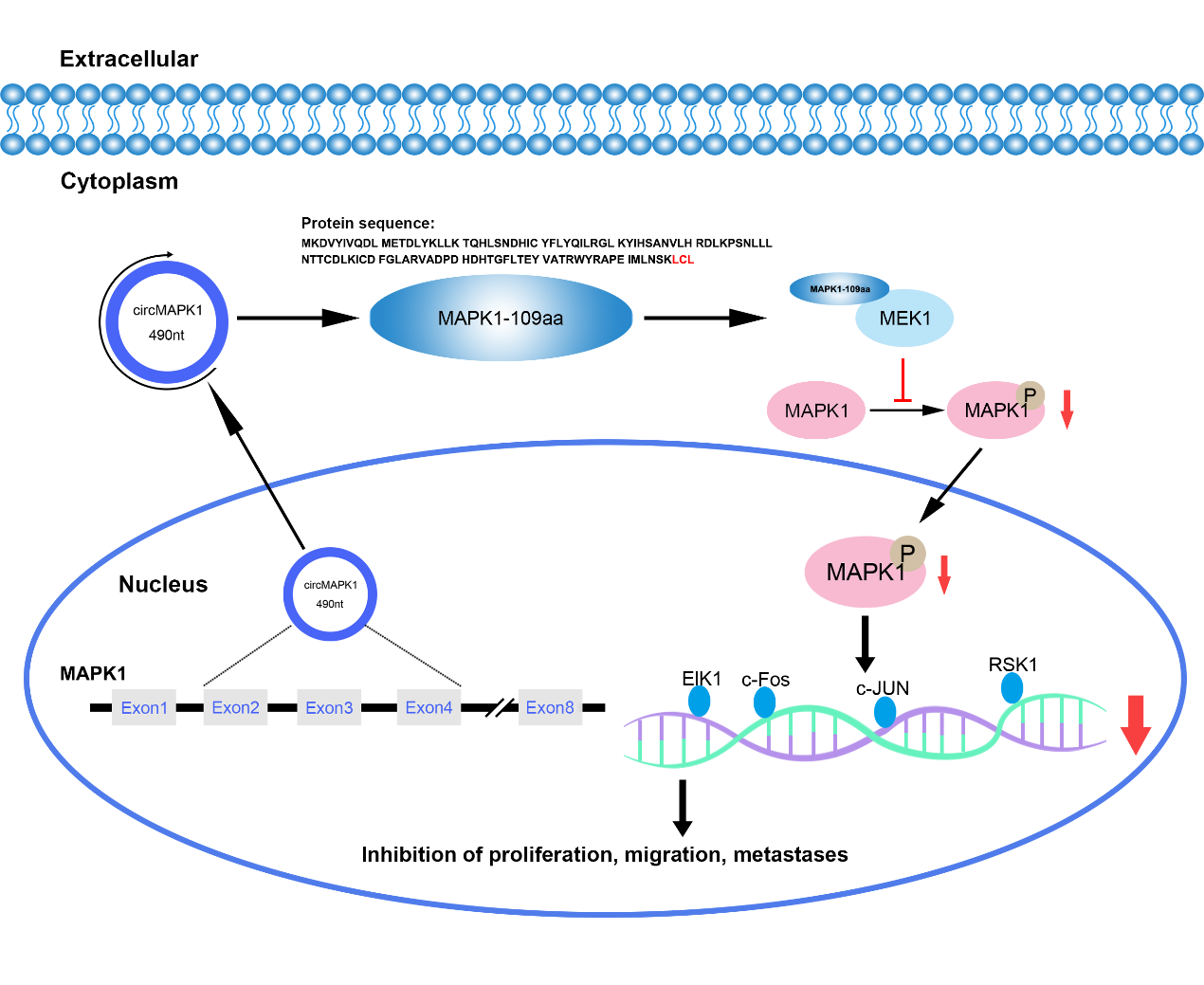


The mechanism diagram of MAPK1-109aa encoded by circMAPK1 on inhibiting proliferation and metastasis of GC via suppressing MAPK1 signaling pathway.
