# Supplementary material for: A novel protein encoded by circMAPK1 inhibits progression of gastric cancer by suppressing activation of MAPK signaling
Source: Mol Cancer. 2021 Apr 9;20:66. doi: 10.1186/s12943-021-01358-y (PMC8034133; doi:10.1186/s12943-021-01358-y)
Supplement: Supplementary file 4 — Additional file 4. Materials and Methods. [file 12943_2021_1358_MOESM4_ESM.doc]

**Materials and Methods:**

**RNA preparation, treatment with RNAse R, and PCR**

According to the manufacturer's protocol, all RNAs were isolated with TRIzol reagent (Invitrogen) and incubated with 3 U / mg RNAse R (Epicentre Technologies, USA) at 37 ° C for 20 minutes. These RNAs were then reverse transcribed into cDNA using the PrimeScript RT reagent (TaKaRa, RR036A, Japan). Quantitative real-time reverse transcription polymerase chain reactions were performed on the 7500 Real-time PCR System (Applied Biosystems, Carlsbad, California, USA) using the Universal SYBR Green Master Mix (4,913,914,001, Roche, Shanghai, China). At the same time, GAPDH was used as an internal reference for circRNA and mRNA, respectively.

**Western blot**

The protein extraction kit (Key Gene, China) was used to extract proteins from stable transfected cells according to the manufacturer's protocol. Protein concentrations were measured using BCA kits (Pierce, Rockford, IL), protein samples were separated by electrophoresis using SDS-containing polyacrylamide gels, and the separated protein samples were placed on a polyvinylidene fluoride (PVDF) membrane (Millipore, Billerica, MA, USA). After blocking with 5% BSA in TBST buffer for 2 hours, the membrane was incubated with the primary antibody overnight at 4 ° C. The membrane was then washed 3 times with TBST buffer for 10 minutes. Membranes were incubated for 2 hours at room temperature using the corresponding HRP-labeled secondary antibody and washed 3 times with TBST buffer. Finally, blot signals were visualized by an enhanced chemiluminescence detection system with a chemiluminescent HRP substrate (Millipore, WBKL0100).

**5-Ethynyl-2′-deoxyuridine (EdU) assay**

The EdU analysis kit (RiboBio, China) was used to detect DNA synthesis and cell proliferation. 100,000 treated GC cells were seeded overnight in a 24-well plate. The next day, Edu solution (10 μM) was added to a 24-well plate and waited for 24 hours. Next, 4% formalin was applied and the GC cell was fixed at room temperature for 2 hours. In the next step, GC cells were infiltrated with 0.5% Triton X-100 for 10 minutes, then Apollo reaction solution (200 μL) was added to stain EdU for 30 minutes, and Hoechst 33342 (200 μL) was added to stain the nucleus. Finally, a Nikon microscope (Nikon Japan) was used to observe DNA synthesis and cell proliferation reflected by the red and blue signals, respectively.

**CCK-8 assay**

The proliferation rate of gastric cells was detected by the Cell Counting Kit-8 assay (Dojindo Laboratories, Kumamoto, Japan). Ten thousand cells were seeded in a 96-well plate, and 10μL of CCK-8 solution was added to each well at the same time daily. After incubation for 2 hours, the absorbance of the experimental wells at 450 nm was measured by an automatic microplate reader (BioTek, Winooski, VT, USA).

**Colony formation assay**

The treated GC cells were seeded in a 6-well plate (1000 cells per well) for 2 weeks. Then the cells in the plate were fixed with 2 mL of methanol for 30 minutes and stained with crystal violet for 20 minutes.

**Transwell assay**

We placed transwell assay inserts (Millipore, Billerica, MA, USA) in a 24-well plate. In the experiment, 600μl of serum-free RPMI 1640 with 10% FBS were first placed in the bottom chamber. Following this, 20,000 cells in 200μl of RPMI 1640 were seeded in the upper chamber. After 24 to 48 h, the cells in the membrane were fixed with methanol and stained with crystal violet. Finally, the cells were observed by a fluorescent inverted microscope.

**Wound-healing assay**

The treated cells were seeded in a 6-well plate and cultured at 37 ° C. When the cells had adhered to the bottom of the plate, a sterile pipette tip was used to create thin scratches of constant width along the center of each hole. Then we photographed images using an inverted microscope (Olympus Optics Co., Ltd., Tokyo, Japan) (0 hours) and marked the 6-well plate so that the same field can be found again. Then the serum culture medium was added to the 6-well plate. After incubating the cells at 37 ° C for 24 hours, we removed the culture medium and washed the cells 3 times with PBS to remove surrounding cellular debris.

**Immunofluorescence analysis**

GC cell lines were seeded on collagen-coated glass and incubated overnight in RPMI 1640 medium at 37 °C in a humidified atmosphere of 5% CO2. The cells were washed twice with PBS, fixed with 4% formaldehyde, and permeabilized with 0.2% Triton X-100. After blocking with 1% BSA for 30 minutes, cells were incubated overnight at 4 ° C with certain primary antibodies. Secondary antibody Cy ™ 3-Goat Anti-Rabbit IgG (Jackson, 1: 100) and DAPI have been added to a specially designed Petri dish. After the final treatment, observe the cells with a confocal microscope (Nikon, Japan).

**Dual-luciferase reporter assay**

The wild-type or mut-IRES sequence was constructed and inserted into the luciferase reporter gene of the circMAPK1 plasmid in HEK293 T cells. After 48h of transfection, luciferase reporter assays were conducted using a dual-luciferase reporter assay system (Promega, Madison, WI) according to the manufacturer’s instructions. Relative luciferase activity was normalized to Renilla luciferase activity.

**Mass spectrometry analysis**

Proteins were separated by SDS-PAGE, gel bands were manually cut and digested with sequencing grade trypsin (Promega, Madison, Wisconsin, USA). Digested peptides were analyzed on a QExactive mass spectrometer (Thermo Fisher Scientific, Waltham, Massachusetts, USA). Fragment spectra were analyzed using the National Center for Biotechnology Information (Matrix Science, Boston, Massachusetts, USA) non-redundant protein database (using Mascot).

**Animal study**

The 5-week-old BALB / c nude mice were purchased from the Laboratory Animal Center of Nanjing Medical University. Nude mice were subcutaneously injected with 200 μL of 5 × 10^6^ transfected cells. Subcutaneous tumors were measured weekly and collected after 4 weeks. Tumor weights and volumes were measured.

Transfected luciferase-labeled cells were intravenously injected into the tail vein of BALB / c nude mice for vivo bioluminescence imaging. Five weeks later, an IVIS imaging system (Caliper Life Sciences, Hopkinton, Mass.) was used to observe distance metastases after fluorescein injection (Caliper Life Sciences, Waltham, Mass.).

**Reagents and antibodies**

Regarding the primary antibodies used in the study, anti-GAPDH( AF5009 ) was purchased from Beyotime (Shanghai, China); anti-MAPK1-109aa (NBP1-87943) was purchased from Novus(Beijing, China); anti-MEK1(12671), anti-Erk1/2 (4695), anti-p-ELK1(9181), anti-p-c-Fos(5348）,anti-p-c-JUN（3270）,anti-p-RSK1（9341）were purchased from Cell Signaling Technology (Danvers, PA USA). MAPK pathway inhibitor（PD0325901 ）and MAPK pathway activator (C16-PAF) were purchased from MedChemExpress (Shanghai, China).

**Statistics**

The experiment was performed 3 times and the results were expressed as mean ± standard deviation. We statistically analyzed the data through Student's t-test using SPSS statistical software and p <0.05 was considered statistically significant. * Means p <0.05, ** means p <0.01, *** means p <0.001.
